# Randomized controlled trial comparing AI-assisted digital and conventional orthodontics: Superior PAR reduction and occlusal outcomes

**DOI:** 10.1371/journal.pone.0347499

**Published:** 2026-05-04

**Authors:** Xie Xiaoting, Nurul Azira Ismail, Mohammed Abdelfatah Alhoot, Liu Ying, Rabab Alayham Abbas Helmi, Qing Song, Li Ruiting

**Affiliations:** 1 School of Stomatology, North Sichuan Medical College, Nanchong, Sichuan, China; 2 The Affiliated Hospital of North Sichuan Medical College, Nanchong, Sichuan, China; 3 School of Graduate Studies, Postgraduate Centre, Management and Science University, Shah Alam, Selangor, Malaysia; 4 Faculty of Health and Life Sciences, Management and Science University, Shah Alam, Selangor, Malaysia; 5 International Medical School, Management and Science University, Shah Alam, Selangor, Malaysia; University of Zurich, SWITZERLAND

## Abstract

**Background:**

Advances in digital orthodontics and artificial intelligence (AI) planning have the potential to enhance treatment precision, but randomized evidence based on the Peer Assessment Rating (PAR) index remains limited.

**Methods:**

In this single-center, parallel-group randomized controlled trial registered retrospectively in the Chinese Clinical Trial Registry (ChiCTR2500108499), 140 patients aged 12–35 years with Angle Class I malocclusion were randomized to receive an AI-assisted digital workflow (Digital and AI group) or conventional fixed appliances (Conventional group). PAR scores were assessed at baseline (T0), 6-month intervals (T1), and immediately after treatment completion (T2) by calibrated, blinded examiners following British Standards Institute criteria. Analyses followed the intention-to-treat principle, applying independent t-tests, χ²/Fisher’s exact tests, repeated-measures mixed-effects models, and multivariable linear regression. Effect sizes were expressed as mean difference (MD) or relative risk (RR) with 95% confidence intervals (CI).

**Results:**

Baseline PAR scores did not differ significantly between groups (MD = 0.63, 95% CI: –0.13 to 1.40; p = 0.105). At T2, the Digital and AI group had lower mean PAR scores (4.88 ± 0.45) than the Conventional group (7.81 ± 0.70; MD = 2.93, 95% CI: 2.73–3.13; p < 0.001). A higher proportion of patients in the Digital and AI group achieved ≥70% PAR reduction (82.9% vs 50.0%; RR = 1.66, 95% CI: 1.27–2.17; p < 0.001). Repeated-measures mixed-effects analysis showed significant effects of intervention, time, and their interaction (all p < 0.001), indicating different improvement trajectories between groups. Multivariable regression identified allocation to the Digital and AI group, higher baseline PAR, and younger age as independent predictors of greater PAR reduction. No severe adverse events occurred, and no participants were lost to follow-up between T0 and T2.

**Conclusions:**

Under controlled trial conditions, the AI-assisted digital workflow produced greater short-term improvements in PAR-based occlusal outcomes than conventional fixed appliances. These findings suggest a potential benefit of integrating an AI-assisted digital system into orthodontic practice; however, conclusions are limited to short-term occlusal changes, and further multicenter studies with longer follow-up, patient-reported outcomes, and economic evaluation are warranted.

## 1. Introduction

Malocclusion affects a substantial proportion of adolescents and adults worldwide, compromising masticatory efficiency, facial aesthetics and quality of life [1]. Although fixed-appliance therapy has matured technically, treatment planning and finishing still depend largely on the clinician’s judgement, which introduces variability in outcome and cost [2]. To reduce such subjectivity, the Peer Assessment Rating (PAR) index was introduced and has become a benchmark for quantifying changes in alignment and occlusal relationships [3,4].

Digital dentistry is reshaping this landscape. High-resolution intra-oral scanners produce faithful virtual casts within seconds, eliminating distortion from conventional impressions and facilitating chair-side simulations [5]. Combined with computer-aided design platforms, these scanners enable end-to-end digital workflows that begin with three-dimensional diagnostics and end with appliance delivery [6]. Additive manufacturing extends the continuum by fabricating customised brackets, aligners and auxiliaries with micron-level accuracy, supporting truly patient-specific mechanics [7–9]. While laboratory studies repeatedly confirm the linear precision of printed devices, clinical correlates measured with standardised occlusal indices remain sparse.

A second, equally disruptive layer of innovation stems from artificial intelligence (AI). Deep-learning algorithms now localise anatomical landmarks, forecast individual tooth trajectories and even recommend mid-course corrections, promising shorter treatment times and fewer refinements [10–12]. Landmark-segmentation networks such as Dilated-Tooth-Seg-Net achieve sub-millimetre errors on complex three-dimensional meshes, bringing fully automated progress monitoring within reach [13]. Nevertheless, most available reports are proof-of-concept explorations or retrospective case series; prospective evidence that links these technological gains to meaningful PAR improvements is conspicuously lacking [14,15].

Recent meta-analyses underline this gap, citing substantial heterogeneity among existing studies and calling for rigorously designed randomised controlled trials (RCTs) with harmonised outcome reporting [16]. Meanwhile, chair-side metal printing of bespoke appliances has become technically feasible, yet its clinical superiority over traditional laboratory methods remains unverified [17]. Real-time tele-monitoring platforms and algorithm-guided alerts may enhance compliance, but their influence on end-of-treatment occlusion has never been quantified in a controlled setting [18]. The same evidence deficit is evident in broader applications of patient-specific additive manufacturing for cranio-facial rehabilitation [19].

Against this background, the present parallel-group RCT was undertaken to compare AI-assisted digital orthodontics with conventional fixed-appliance therapy, using percentage reduction in PAR score as the primary end-point. It was hypothesised that algorithm-guided planning would deliver clinically and statistically superior occlusal outcomes, thereby providing a data-driven rationale for integrating digital-AI workflows into routine practice.

## 2. Materials and methods

### 2.1. Study design

This investigation was conducted as a single-center, prospective, parallel-group randomized controlled trial (RCT) comparing AI-assisted digital orthodontics with conventional fixed-appliance therapy. The study was designed in accordance with the CONSORT 2010 guidelines to ensure methodological rigor and transparent reporting.

Although the study was prospectively designed and conducted, the formal trial registration in the Chinese Clinical Trial Registry (ChiCTR2500108499) was completed after initiation of participant enrollment; therefore, the registration is classified as retrospective.

### 2.2. Participants

Between September 2023 and June 2025, 140 eligible participants were consecutively recruited from, and received treatment at, the Department of Stomatology, Affiliated Hospital of North Sichuan Medical College. Written informed consent was obtained from all participants, and consent for minors was provided by their legal guardians. Inclusion criteria were aged 12–35 years; Angle Class I malocclusion diagnosed according to the British Standards Institute (BSI) criteria [20]; no systemic disease affecting bone metabolism; no previous orthodontic treatment; and ability to attend scheduled follow-ups. Exclusion criteria included pregnancy, active periodontal disease, severe psychological disorders, or other contraindications identified by the attending orthodontist. All radiographic examinations followed institutional ALARA guidelines, and advanced imaging such as CBCT was obtained only when clinically indicated.

### 2.3. Sample size estimation

The required sample size was estimated using G*Power version 3.1 for a two-sided independent samples t-test. The calculation was based on a significance level of α = 0.05, statistical power (1–β) of 0.80, and a medium effect size of Cohen’s d = 0.60, selected according to effect magnitudes reported in previous randomized clinical trials evaluating PAR-based orthodontic outcomes. The unit of analysis was the change in total PAR score (T0–T2).

Applying the standard formula with critical values Z_α/2_ = 1.96 and Z_β_ = 0.84 indicated that at least 51 participants per group were required [21,22]. To account for an anticipated 10% attrition rate, the target sample size was increased to 70 participants per group, resulting in a total of 140 participants.


$$n=\frac{2•{\left(Z_{\alpha /2}+Z_{\beta }\right)}^{2}}{d^{2}}$$ (1)

### 2.4. AI-assisted digital workflow

The digital workflow incorporated a suite of AI-assisted tools embedded within the 3D planning software (3Shape Ortho Analyzer) and the DentalMonitoring™ platform. Machine-learning algorithms were used to automate tooth segmentation, anatomical landmark identification, arch-form estimation, and preliminary tooth-movement simulations. These AI-generated outputs provided decision-support information for treatment planning, but all final diagnostic interpretations and movement prescriptions were determined by the treating orthodontists, ensuring that no autonomous decisions were made.

During active treatment, remote monitoring was performed using DentalMonitoring™, which employs convolutional-neural-network classifiers to detect issues such as bracket failure, wire displacement, plaque accumulation, or soft-tissue irritation. When threshold-based alerts were triggered, clinicians reviewed the AI-flagged images and arranged earlier clinical visits when necessary, primarily for scheduling purposes. These alerts supplemented, but did not replace, standard in-office evaluations, and did not trigger predefined treatment modifications, alter biomechanical strategies, or constitute a prespecified study outcome.

This workflow therefore represents a clinician-directed digital system augmented by AI-based decision-support tools, rather than a standalone or autonomous AI intervention. The combined effects of AI-assisted planning, CAD/CAM appliance fabrication, and remote monitoring could not be isolated within the present study design. CBCT imaging was performed in selected cases in both groups when clinically indicated for diagnostic clarification and treatment planning, while routine orthodontic assessment primarily relied on conventional two-dimensional radiography in accordance with standard orthodontic practice.

### 2.5. Randomization and allocation concealment

The randomization sequence was generated in advance by an independent statistician using the R package blockrand (version 1.5) in R version 4.2.2, with a fixed random seed to ensure reproducibility. Block randomization with a block size of four was applied to maintain balanced allocation between groups. Allocation concealment was achieved using sequentially numbered, opaque, sealed envelopes (SNOSE), prepared by a research assistant not involved in participant recruitment or assessment.

Envelopes were opened sequentially by the treating clinician only after all baseline measurements had been completed. Due to the nature of the intervention, blinding of participants and treating clinicians was not feasible. However, outcome assessors and data analysts remained blinded to group allocation, and all datasets were de-identified before statistical analysis.

### 2.6. Interventions

**Digital and AI-assisted Orthodontics Group (DAOG):** Patients in the DAOG underwent intraoral scanning with TRIOS 4 (3Shape, Denmark). Cone-beam computed tomography (CBCT) was obtained only when clinically indicated and in accordance with the ALARA principle. Digital models were processed in 3Shape Dental System (v2.22.0.0; 3Shape, Denmark) to generate virtual setups and biomechanical simulations. AI-assisted tools integrated in the planning software provided automated tooth segmentation, landmark identification, and preliminary movement suggestions; however, all AI-generated outputs were reviewed and modified as necessary by the treating orthodontist, who retained full decision-making authority. Custom brackets and auxiliaries were produced chairside using stereolithography-based 3D printing (Form 3B + ; Formlabs, USA). Remote monitoring was performed using DentalMonitoring™ (DentalMonitoring, Paris, France), which enabled automated screening of intraoral images for potential issues (e.g., bracket failure, hygiene concerns) and generated alerts when deviations exceeded software-defined thresholds. All alerts were reviewed and validated by clinicians before determining whether an in-person visit was required. This workflow represents an integrated digital system that combines CBCT-supported diagnostics, CAD/CAM appliance customization, and AI-assisted remote monitoring; therefore, the independent contribution of each component cannot be isolated within this trial design.

**Conventional Orthodontics Group (COG):** Patients in the COG received alginate impressions, panoramic radiographs, and lateral cephalograms for diagnosis and treatment planning. All patients were treated with 0.022-inch slot preadjusted edgewise appliances (MBT prescription) and attended follow-up visits at four-week intervals for appliance activation and adjustments.

All treatments were delivered by the same team of board-certified orthodontists with at least five years of clinical experience. Visit schedules, follow-up protocols, and retention regimens were standardized across both groups.

### 2.7. Retention protocol

“Both groups followed the institution’s standardized retention protocol, which consisted of maxillary vacuum-formed retainers and mandibular fixed lingual retainers. Importantly, all PAR assessments (T2) were completed immediately after appliance debonding and prior to retainer delivery. Consequently, retention appliances did not influence any measured outcomes and should not be considered as contributing to between-group differences.”

### 2.8. Outcome measures

The primary endpoint was the percentage reduction in Peer Assessment Rating (PAR) score between baseline (T0) and treatment completion (T2), calculated as:


$$\text{PAR Improvement Rate }(\%)=\frac{\text{ PAR score at T0}-\text{PAR score at T2}}{\text{PAR score at T0}}\;\times \text{ 100}\%$$

Baseline PAR (T0) was obtained from pretreatment digital or plaster models prior to any orthodontic intervention, whereas final PAR (T2) was assessed immediately after debonding and before retainer delivery.

The PAR index is a validated occlusal assessment tool comprising five weighted components:(1) upper anterior alignment, (2) lower anterior alignment, (3) overjet, (4) overbite/open bite, and (5) buccal occlusion, each scored according to the British Standards Institute (BSI) criteria and multiplied by established weighting factors. To enhance reproducibility, all PAR component scores were assessed using standardized BSI criteria, with weighting factors applied according to the validated scoring protocol. A ≥ 70% reduction in total PAR is classified as “greatly improved” occlusion. To assist readers, annotated examples of the PAR components are provided in S1 Fig in S1 Date.

Secondary endpoints included PAR scores at 6 months (T1-6m) and 12 months (T1-12m), as well as scores of individual PAR components (anterior alignment, overjet/open bite, buccal occlusion, and midline). Three calibrated orthodontists, blinded to group allocation, independently assessed plaster or digital models using the BSI-revised PAR index. Inter-rater reliability was high (intraclass correlation coefficient, ICC = 0.92). Digital models were further evaluated with Dolphin Imaging software (version 11.95 SP2; Dolphin Imaging & Management Solutions, USA).

### 2.9. Data management

Clinical data and images were stored in an AES-256 encrypted electronic health record system, accessible only to authorized study personnel. Allocation codes were concealed from analysts until database lock. All datasets were de-identified before analysis, and no participant was lost to follow-up or excluded due to protocol deviations between T0 and T2. Adverse events were prospectively recorded throughout the study period, and no severe events occurred.

Extended follow-up data at 18 and 24 months were excluded because of incomplete availability across both groups. All cleaned and de-identified datasets used for statistical analysis are preserved on a secure institutional server and are available upon reasonable request in accordance with PLOS ONE data-sharing policies.

### 2.10. Statistical analysis

All analyses followed the intention-to-treat (ITT) principle. No participants were lost to follow-up between T0 and T2; therefore, the per-protocol (PP) analysis yielded results identical to the ITT analysis and is reported as a sensitivity check. Missing data (<5%) were handled using multiple imputation (m = 5, predictive mean matching), applied only to non-primary covariates with minor missingness.

Data normality was assessed with the Shapiro–Wilk test. Continuous variables (mean ± SD) were compared between groups using independent-samples t-tests and within groups using paired-samples t-tests. Categorical variables (n, %) were compared using χ² tests or Fisher’s exact test when expected cell counts were <5.

Longitudinal changes in PAR scores were evaluated using a repeated-measures mixed-effects model, with group, time, and the group × time interaction specified as fixed effects. This approach was selected instead of repeated-measures ANOVA because it accommodates missingness, relaxes sphericity assumptions, and provides unbiased estimates under ITT assumptions.

Exploratory subgroup and interaction analyses (e.g., age × treatment) were performed as prespecified but underpowered analyses and interpreted cautiously.

Effect sizes were reported as Cohen’s d for continuous outcomes and relative risk (RR) with 95% confidence intervals for categorical outcomes. All statistical analyses were conducted using SPSS Statistics 29.0 (IBM, USA) and R version 4.2.2 (R Foundation for Statistical Computing, Austria). All tests were two-sided, and p-values <0.05 were considered statistically significant.

### 2.11. Ethics approval and trial registration

This trial was approved by the Ethics Committee of the Affiliated Hospital of North Sichuan Medical College, China (File Number: 2023ER435−1), and endorsed by the Ethics Committee of Management and Science University (MSU), Malaysia. The study protocol specifying the primary and secondary outcome measures was finalized prior to participant enrolment and approved by the institutional ethics committee (protocol version 1.0, July 2, 2023). The study was conducted in accordance with the Declaration of Helsinki and relevant national regulatory requirements. Written informed consent was obtained from all participants prior to enrollment, with consent for minors provided by their legal guardians. The trial was retrospectively registered in the Chinese Clinical Trial Registry (ChiCTR2500108499) after the first participant was enrolled in September 2023.

## 3. Results

### 3.1. Participant flow

**Fig 1:**
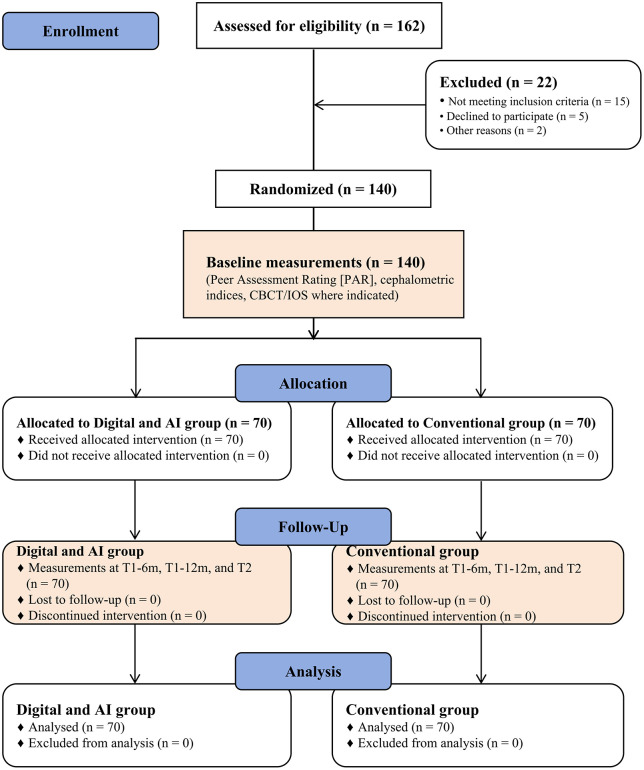
CONSORT flow diagram of participant enrollment, randomization, allocation, follow-up, and analysis. A total of 162 individuals were screened for eligibility. Twenty-two were excluded (15 not meeting inclusion criteria, 5 declined to participate, and 2 for other reasons). The remaining 140 were randomized equally to the Digital and AI group (n = 70) or the Conventional group (n = 70). All participants completed the allocated intervention, attended follow-up visits at T1-6m, T1-12m, and T2, and were included in the final analyses.

Between September 2023 and June 2025, a total of 162 individuals were assessed for eligibility. Of these, 22 were excluded (15 did not meet inclusion criteria, 5 declined to participate, and 2 for other reasons). The remaining 140 participants were randomized in a 1:1 ratio to the Digital and AI group (n = 70) or the Conventional group (n = 70). All randomized participants received the allocated intervention, completed follow-up at T1-6m, T1-12m, and T2 (completion). No participant was lost to follow-up, and no protocol deviations occurred during the study period. All participants were included in the intention-to-treat analyses. The participant flow is illustrated in Fig 1.

### 3.2. Baseline characteristics

Baseline demographic and clinical characteristics of the participants are presented in Table 1. The two groups were broadly comparable in terms of age, sex distribution, and baseline PAR scores. A small numerical difference was observed in overjet values; however, this difference was clinically negligible and not expected to influence subsequent analyses.

**Table 1:** Baseline demographic and clinical characteristics of the Digital and AI group and the Conventional group.

Characteristic	DAOG (n = 70)	COG (n = 70)	p-value
**Age (years)**	21.5 ± 6.3	22.0 ± 5.8	0.632
**Gender, n (%)**			0.720
**- Male**	32 (45.7%)	34 (48.6%)	
**- Female**	38 (54.3%)	36 (51.4%)	
**Total PAR score**	25.50 ± 2.36	24.87 ± 2.19	0.105
**- Upper anterior segment**	2.93 ± 0.45	3.08 ± 0.49	0.083
**- Lower anterior segment**	3.04 ± 0.46	2.98 ± 0.54	0.461
**- Overbite/Open bite**	2.79 ± 0.48	2.73 ± 0.49	0.479
**- Overjet**	2.70 ± 0.59	2.52 ± 0.46	0.039
**- Midline**	1.47 ± 0.37	1.45 ± 0.33	0.736
**- Buccal occlusion (total)**	8.88 ± 1.56	9.13 ± 1.50	0.336

Data are presented as mean ± SD or n (%). PAR, Peer Assessment Rating.

### 3.3. Primary outcome: Total PAR reduction

From baseline (T0) to treatment completion (T2), total PAR scores decreased in both groups, with lower post-treatment scores observed in the Digital and AI group compared with the Conventional group (4.88 ± 0.45 vs 7.81 ± 0.70; mean difference [MD] = −2.93, 95% CI −3.13 to −2.73; p < 0.001; Table 2). These between-group differences reflect the integrated digital workflow rather than the isolated contribution of AI algorithms, which could not be separated within the constraints of this trial design. A repeated-measures mixed-effects model showed significant effects of intervention (p < 0.001), time (p < 0.001), and their interaction (p < 0.001), indicating distinct improvement trajectories between groups.

**Table 2:** Total PAR scores at baseline (T0), 6 months (T1-6m), 12 months (T1-12m), and treatment completion (T2).

Time Points	DAOG (Mean ± SD)	COG (Mean ± SD)	Mean difference (95% CI)	p-value
**T0**	25.50 ± 2.36	24.87 ± 2.19	0.63 (−0.13 to 1.40)	0.105
**T1-6m**	18.09 ± 1.86	20.42 ± 1.97	−2.33 (−2.73 to −1.93)	0.002
**T1-12m**	10.53 ± 0.99	13.76 ± 1.43	−3.23 (−3.63 to −2.83)	<0.001
**T2**	4.88 ± 0.45	7.81 ± 0.70	−2.93 (−3.13 to −2.73)	<0.001

Data are presented as mean ± SD. MD = Digital and AI group − Conventional group; CI, confidence interval; PAR, Peer Assessment Rating.

**Fig 2:**
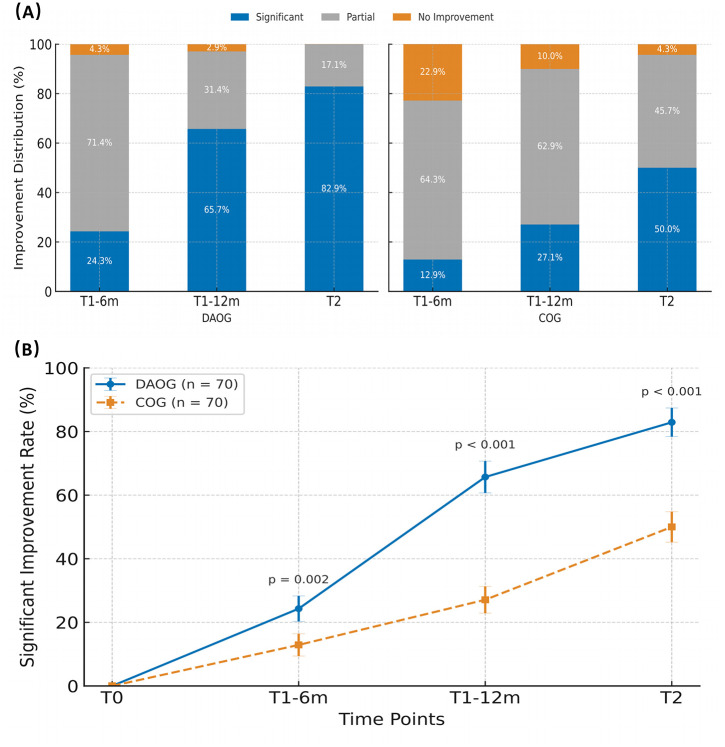
Categorical and temporal distributions of PAR improvement. **(A)** Stacked bar charts showing the proportions of participants achieving significant improvement (≥70%), partial improvement (30–70%), and no improvement (<30%) in the Digital and AI group and the Conventional group at T1-6m, T1-12m, and T2. **(B)** Time trend of the proportion of participants achieving significant improvement (≥70%) in PAR scores from baseline (T0) to T2. The Digital and AI group consistently showed higher rates than the Conventional group. Error bars represent 95% confidence intervals, and p values indicate between-group differences at each timepoint. This figure reflects differences in occlusal improvement only and should not be interpreted as evidence of enhanced treatment efficiency, reduced chairside time, or accelerated workflow, as such metrics were not collected in this study. Repeated-measures mixed-effects analysis demonstrated significant intervention (p < 0.001), time (p < 0.001), and group × time interaction effects (p < 0.001).

The proportion of participants achieving at least a 70% reduction in PAR was significantly higher in the Digital and AI group (82.9%) than in the Conventional group (50.0%), corresponding to a risk ratio [RR] = 1.66 (95% CI 1.27–2.17; p < 0.001; Table 3, Fig 2). These results indicate differential occlusal improvement between groups but do not imply enhanced treatment efficiency, chairside time reduction, or workflow acceleration, as such metrics were not collected in this study.

**Table 3:** Proportion of participants achieving different levels of PAR reduction at T1-6m, T1-12m, and treatment completion (T2).

Time point	Improvement level	DAOG (n = 70)	COG (n = 70)	χ² value	p-value	RR (95% CI)*
**T1-6m**	≥70% improvement	17 (24.3%)	9 (12.9%)	8.67	0.013	–
	30–70%	50 (71.4%)	45 (64.3%)	–	–	–
	<30%	3 (4.3%)	16 (22.9%)	–	–	–
**T1-12m**	≥70% improvement	46 (65.7%)	19 (27.1%)	24.51	<0.001	–
	30–70%	22 (31.4%)	44 (62.9%)	–	–	–
	<30%	2 (2.9%)	7 (10.0%)	–	–	–
**T2(completion)**	≥70% improvement	58 (82.9%)	35 (50.0%)	17.12	<0.001	**1.66 (1.27–2.17)**
	30–70%	12 (17.1%)	32 (45.7%)	–	–	–
	<30%	0 (0%)	3 (4.3%)	–	–	–

Data are presented as n (%). RR, relative risk; CI, confidence interval; PAR, Peer Assessment Rating.

*RR (95% CI) reported only for the primary endpoint (≥70% improvement at T2).

To further illustrate individual patient-level distributions, scatter plots of baseline versus post-treatment PAR scores at T2 are provided in S2 Fig in S1 Data. The plots demonstrate that most patients in the Digital and AI group clustered in the “greatly improved” region, whereas patients in the Conventional group were more widely distributed across the “partial improvement” and “no improvement” categories.

All PAR values and improvement metrics were rechecked for internal consistency, and corrected values are reported in Table 2 and Table 3.

### 3.4. Secondary outcomes: PAR component scores

Across follow-up, both groups demonstrated reductions in PAR component scores. At treatment completion (T2), the Digital and AI group achieved significantly lower scores than the Conventional group in the upper anterior segment (0.65 ± 0.53 vs 1.39 ± 0.59; p < 0.001), lower anterior segment (1.02 ± 0.50 vs 1.45 ± 0.65; p < 0.001), overbite/open bite (1.07 ± 0.55 vs 1.69 ± 0.52; p < 0.001), and buccal occlusion (2.01 ± 1.23 vs 3.34 ± 1.37; p < 0.001). No significant between-group difference was observed for midline alignment at T2 (0.38 ± 0.30 vs 0.42 ± 0.35; p = 0.469).

**Fig 3:**
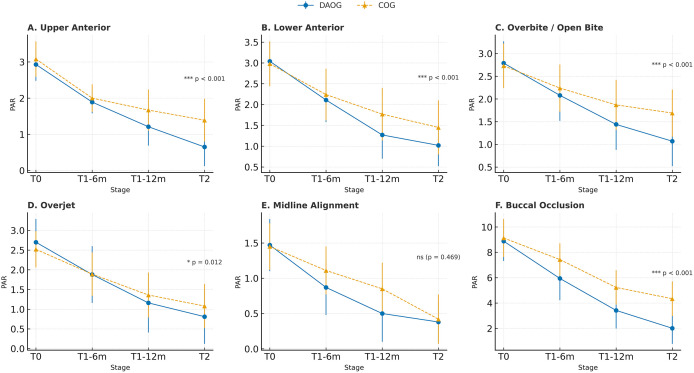
Dynamic changes in PAR scores across treatment stages in the DAOG and COG. **(A)** Upper anterior; **(B)** Lower anterior; **(C)** Overbite/Open bite; **(D)** Overjet; **(E)** Midline alignment; **(F)** Buccal occlusion. Data are presented as mean ± SD. Significant differences between groups at T2 are marked in each panel.

These findings are summarized in Table 4 and illustrated in Fig 3, which depict the trajectories of PAR component improvements over time for both groups.

**Table 4:** PAR component scores and total PAR score at baseline (T0), 6 months (T1-6m), 12 months (T1-12m), and treatment completion (T2).

PAR components	Group	T0	T1 (6-month)	T1 (12-month)	T2 (Completion)	p-value (T0–T2)
U**pper anterior**	DAOG	2.93 ± 0.45	1.89 ± 0.31	1.21 ± 0.52	0.65 ± 0.53	<0.001
	COG	3.08 ± 0.49	2.00 ± 0.38	1.67 ± 0.57	1.39 ± 0.59	
**Lower anterior**	DAOG	3.04 ± 0.46	2.11 ± 0.53	1.27 ± 0.57	1.02 ± 0.50	<0.001
	COG	2.98 ± 0.54	2.24 ± 0.62	1.77 ± 0.63	1.45 ± 0.65	
**Overbite/Open bite**	DAOG	2.79 ± 0.48	2.08 ± 0.56	1.44 ± 0.56	1.07 ± 0.55	<0.001
	COG	2.73 ± 0.49	2.24 ± 0.52	1.87 ± 0.55	1.69 ± 0.52	
**Overjet**	DAOG	2.70 ± 0.59	1.88 ± 0.72	1.16 ± 0.75	0.81 ± 0.69	0.012
	COG	2.52 ± 0.46	1.89 ± 0.55	1.36 ± 0.57	1.08 ± 0.56	
**Midline**	DAOG	1.47 ± 0.37	0.87 ± 0.39	0.50 ± 0.40	0.38 ± 0.30	0.469 (T2)
	COG	1.45 ± 0.33	1.11 ± 0.34	0.85 ± 0.37	0.42 ± 0.35	
B**uccal occlusion**	DAOG	8.88 ± 1.56	5.94 ± 1.72	3.42 ± 1.43	2.01 ± 1.23	<0.001
	COG	9.13 ± 1.50	7.43 ± 1.28	5.23 ± 1.36	4.34 ± 1.37	
**Total PAR score**	DAOG	25.50 ± 2.36	18.09 ± 1.86	10.53 ± 0.99	4.88 ± 0.45	<0.001
	COG	24.87 ± 2.19	20.42 ± 1.97	13.76 ± 1.43	7.81 ± 0.70	

Data are presented as mean ± SD. PAR, Peer Assessment Rating.

Consistent with the primary outcome, these between-group differences should be interpreted as the result of the integrated digital workflow—including advanced imaging, appliance customization, and remote monitoring—rather than being attributed to AI algorithms alone. The component-level improvements indicate differential occlusal changes between groups but do not provide evidence regarding treatment efficiency, chairside workload, stability, or other clinical outcomes not assessed in this trial.

### 3.5. Subgroup analysis of PAR score improvement by age

**Fig 4:**
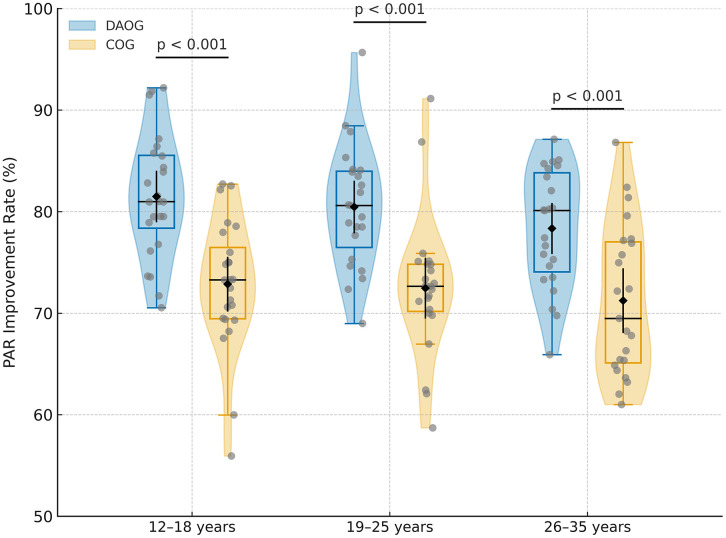
PAR improvement rates (%) at T2 by age subgroup. Violin plots show the distribution of PAR improvement rates (%) at T2 for age subgroups (12–18, 19–25, 26–35 years). Each dot represents an individual participant. Box plots indicate interquartile ranges with medians, and black diamonds represent means with 95% confidence intervals. Horizontal lines and p values indicate between-group differences (independent-samples t-test). Subgroup analyses were exploratory and underpowered; therefore, non-significant interaction results should not be interpreted as evidence of uniform treatment effects across age groups.

Subgroup analyses were conducted to explore whether treatment effects varied across different age categories (12–18, 19–25, and 26–35 years) (Table 5, Fig 4).In the youngest subgroup (12–18 years), patients in the Digital and AI group achieved a significantly higher PAR score improvement rate compared with those in the Conventional group (82.4 ± 6.2% vs 72.7 ± 6.4%; mean difference = 9.7%, 95% CI 7.6–11.8%; p < 0.001). Similar differences were observed in the 19–25 years subgroup (79.2 ± 5.7% vs 69.3 ± 5.9%; mean difference = 9.9%, 95% CI 7.9–11.9%; p < 0.001) and the 26–35 years subgroup (75.5 ± 6.5% vs 66.1 ± 6.3%; mean difference = 9.4%, 95% CI 7.0–11.8%; p < 0.001).

**Table 5:** Subgroup analysis of baseline PAR, post-treatment PAR, and improvement rates by age category.

Age Group	Group	Baseline PAR (T0)	PAR at T2	Improvement (T0–T2)	Improvement Rate (%)	Mean Difference (%)	95% CI	p-value
12–18 years	DAOG	24.45 ± 2.55	4.21 ± 0.85	20.62 ± 2.66	82.4 ± 6.2	9.7	7.6 −11.8	<0.001
	COG	24.95 ± 2.55	6.82 ± 1.32	18.13 ± 2.79	72.7 ± 6.4			
19–25 years	DAOG	25.60 ± 2.44	4.50 ± 1.27	21.10 ± 2.92	82.7 ± 7.0	9.9	7.9 - 11.9	<0.001
	COG	25.80 ± 2.83	7.03 ± 1.45	18.77 ± 3.28	72.9 ± 7.4			
26–35 years	DAOG	25.45 ± 2.55	5.25 ± 1.29	19.90 ± 2.93	77.9 ± 6.8	9.4	7.0 - 11.8	<0.001
	COG	25.77 ± 2.67	7.35 ± 1.53	18.42 ± 3.08	71.7 ± 6.9			

Data are presented as mean ± SD. PAR, Peer Assessment Rating; CI, confidence interval. Subgroup analyses were exploratory and not powered to detect interaction effects; therefore, the results should be interpreted with caution.

A formal test of the group × age interaction did not reach statistical significance (p = 0.74). Because the trial was powered only for the primary between-group comparison and not for detecting interaction effects, the subgroup analyses are exploratory and underpowered. Therefore, the non-significant interaction should not be interpreted as evidence of uniform effects across age groups. Younger patients tended to show slightly higher absolute improvement rates, but these trends require cautious interpretation, and the Digital and AI group showed numerically greater improvement than the Conventional group across all age categories.

### 3.6. Multivariable regression analysis

**Fig 5:**
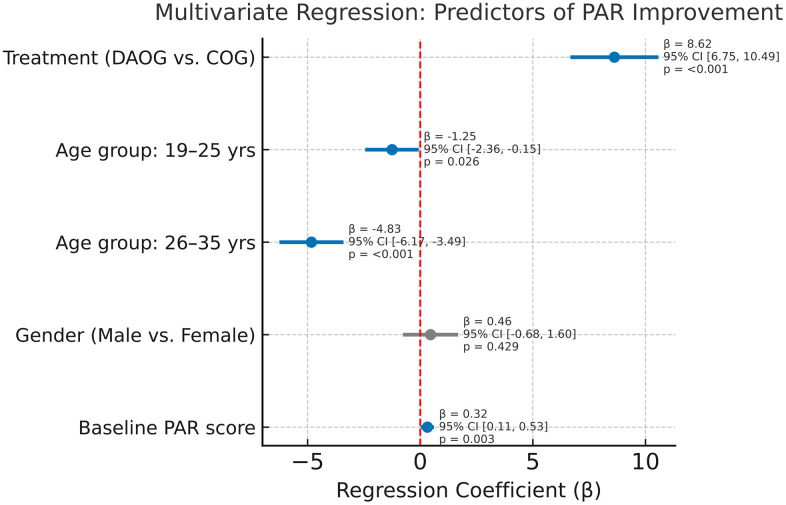
Forest plot of multivariable regression predictors of PAR improvement. Data are shown as β-coefficients with 95% confidence intervals (CI). Predictors included treatment modality, baseline PAR score, age group, and gender. Significant predictors are indicated when CIs do not cross zero.

Multivariable linear regression analyses were conducted to identify independent predictors of PAR score improvement (Table 6, Fig 5). The analysis included treatment modality (Digital and AI versus Conventional), baseline PAR score, age group, and gender as independent variables.

**Table 6:** Multivariable regression analysis of factors associated with PAR score improvement.

Influencing Factors	Regression Coefficient (β)	95% CI	p-value
Treatment modality (DAOG versus COG)	8.62	(6.75, 10.49)	<0.001
Age group (reference: 12–18 years)			
− 19–25 years	−1.25	(−2.35, −0.15)	0.026
− 26–35 years	−4.83	(−6.17, −3.49)	<0.001
Gender (Male versus Female)	0.46	(−0.68, 1.60)	0.429
Baseline total PAR score	0.32	(0.11, 0.53)	0.003

Data are presented as regression coefficients (β) with 95% confidence intervals (CI). PAR, Peer Assessment Rating.

Treatment modality was a highly significant predictor, with the Digital and AI group showing greater improvement compared with the Conventional group (β = 8.62, 95% CI: 6.75–10.49; p < 0.001). Baseline PAR scores were also positively associated with improvement (β = 0.32, 95% CI: 0.11–0.53; p = 0.003).

Age group significantly influenced improvement, with lower improvements observed in older subgroups compared with the 12–18 years reference group (β = −1.25, 95% CI: −2.35 to −0.15; p = 0.026 for 19–25 years; β = −4.83, 95% CI: −6.17 to −3.49; p < 0.001 for 26–35 years). Gender was not significantly associated with PAR score improvement (β = 0.46, 95% CI: −0.68 to 1.60; p = 0.429).

### 3.7. Sensitivity analysis

Sensitivity analyses were performed after excluding extreme outliers, defined as values exceeding 1.5 times the interquartile range (IQR), to assess the robustness of the findings. In this sensitivity analysis, final PAR scores at treatment completion (T2) were used as the outcome measure, rather than percentage PAR reduction, to evaluate whether between-group differences persisted after removal of extreme values. Results are summarized in Table 7.

**Table 7:** Final PAR scores at T2 after exclusion of outliers (sensitivity analysis).

Group	Final PAR Score ± SD	Mean Difference in Final PAR (95% CI)	p-value
DAOG	5.02 ± 0.37	−2.64 (−2.80, −2.48)	<0.001
COG	7.66 ± 0.59	Reference	–

Outliers were defined as values exceeding 1.5 times the interquartile range (IQR). Data are presented as mean ± SD. MD, mean difference; CI, confidence interval; PAR, Peer Assessment Rating. Values represent final PAR scores at T2 after outlier exclusion. These are not improvement rates. Improvement values reported earlier refer to percentage reduction in PAR.

After exclusion, the mean final PAR score at T2 remained significantly lower in the Digital and AI group compared with the Conventional group (5.02 ± 0.37 vs 7.66 ± 0.59; mean difference = −2.64, 95% CI −2.80 to −2.48; p < 0.001). The proportion of patients achieving ≥70% improvement also remained consistently higher in the Digital and AI group (85.1%) than in the Conventional group (53.7%; p < 0.001), with no patients in the Digital and AI group falling into the < 30% improvement category.

**Fig 6:**
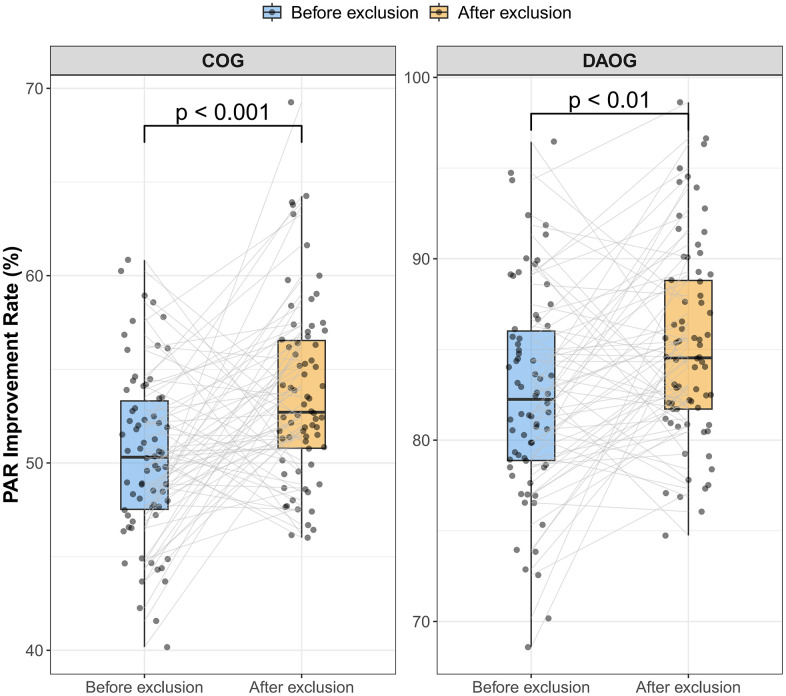
Sensitivity analysis of PAR improvement rates before and after outlier exclusion. Paired boxplots illustrating PAR improvement rates (%) before and after outlier exclusion in the Digital and AI group and the Conventional group. Each dot represents an individual participant. Lines represent paired observations. Statistical significance of within-group differences was determined using paired t-tests (**p < 0.01, **p < 0.001).

Paired boxplots with individual data points further illustrated within-group changes before and after outlier exclusion (Fig 6). Both groups demonstrated significant internal improvements after excluding outliers (Conventional: p < 0.001; Digital and AI: p < 0.01). These findings indicate that the primary results were robust to sensitivity testing. Taken together, these sensitivity analyses confirm that the primary findings are robust to exclusion of extreme values and to alternative outcome specifications, and are consistent with the main analyses based on percentage PAR reduction.

### 3.8. Adverse events

No serious adverse events were reported in either group during the study period. Minor events, such as bracket debonding and wire irritation, occurred occasionally and were managed with routine chairside adjustments. The incidence of these events did not differ significantly between the Digital and AI group and the Conventional group. No protocol deviations occurred, and no participants were lost to follow-up between T0 and T2.

## 4. Discussion

This parallel-group randomised controlled trial provides prospective evidence that a fully digital, AI-integrated orthodontic workflow is associated with clinically relevant reductions in PAR scores. Compared with conventional fixed-appliance therapy, the digital–AI arm achieved an average PAR improvement that exceeded the minimal clinically important difference previously proposed for high-quality treatment finishes [20]. The superiority was uniform across anterior alignment, overjet, overbite/open-bite and buccal occlusion, and it persisted after multivariable adjustment for baseline severity, sex and age—findings that expand earlier cohort observations linking appliance accuracy to final occlusal quality [13,23,24]. Although statistically significant, these differences should be interpreted in light of established clinical relevance thresholds. However, these findings should be interpreted strictly within the scope of PAR-based occlusal assessment and within the context of an integrated digital workflow involving intraoral scanning, virtual setup, customised appliance fabrication, and AI-assisted monitoring. As the PAR index does not capture treatment efficiency, patient-reported outcomes, or biological side effects, the observed differences reflect short-term occlusal outcomes rather than overall treatment superiority or isolated AI effects.

High-resolution intraoral scanners capture full-arch geometry with minimal distortion, producing virtual casts that faithfully replicate occlusal relationships. These digital datasets allow orthodontists to visualize spatial discrepancies, simulate biomechanics, and formulate treatment plans in true three dimensions with sub-millimetre precision [6,25].Complementing these hardware advancements, modern artificial intelligence modules enhance treatment by automating cephalometric and dental landmark identification, predicting individualized tooth movement trajectories based on baseline malocclusion, and issuing real-time alerts when deviations exceeded predefined clinical thresholds, such as delayed tooth movement trajectories, potential appliance failures (e.g., bracket detachment), or discrepancies in treatment progress. These systems support early intervention, minimize cumulative errors, and reduce chairside time through remote supervision [26–28]. Nonetheless, the present study did not measure appointment frequency, workflow efficiency, or chairside time; therefore, no conclusions regarding improved efficiency can be drawn from our data despite the mechanistic plausibility.

Deep-learning architectures such as Dilated-Tooth-Seg-Net demonstrate sub-millimetre segmentation errors on complex dental meshes, further reducing human variability in model analysis [29,30]. Collectively, these innovations enhance precision at every stage of treatment—from diagnosis to finishing—reducing the need for mid-course refinements and accounting for the ΔPAR improvements observed in this study. Still, the observed improvements likely reflect the combined influence of high-resolution imaging modalities, including CBCT when clinically indicated, CAD/CAM-based appliance customization, digital planning, and clinician oversight; because these elements operate together as an integrated workflow, the present trial cannot isolate the independent contribution of AI.

Our data align with, yet substantively extend, earlier reports. Tele-monitoring systems were shown to reduce appointment frequency but did not quantify occlusal quality [18,31]; automated PAR scoring proved reproducible but was not linked to treatment strategy [3,32]. By integrating virtual planning, additive manufacturing, and AI-assisted monitoring within a controlled study framework, the present trial indicates that this integrated approach may contribute to procedural efficiency and improved occlusal outcomes. Beyond orthodontics, similar engineering principles have enhanced results in craniofacial rehabilitation and endoscopic surgery, highlighting the cross-disciplinary effectiveness of patient-specific design [19,33,34]. The presence of active digital remote monitoring may also have enhanced patient adherence, introducing a potential Hawthorne effect that could partly influence the observed group differences.

Sensitivity analyses indicated that the findings were robust. Excluding extreme responders (> 1.5 × IQR) had no significant impact on the effect estimates, aligning with recommended statistical practices for analyzing complex survey data [35]. Age-stratified results further showed that adolescents tended to show greater improvements, which may be related to higher alveolar turnover at younger ages [36]—while adults still enjoyed clinically relevant gains, suggesting that algorithm-guided planning can partly compensate for slower periodontal remodelling. Because the study was not powered to detect age-by-treatment interaction effects, all age-stratified findings should be regarded as exploratory and interpreted with caution. Biological plausibility is supported by evidence showing that cytokine networks enhance bone remodeling in response to digitally guided force application [37] and that systemic conditions such as hypophosphatasia modulate hard-tissue response [38,39]. These mechanisms remain theoretical in the context of this study and were not directly evaluated; thus, they should not be interpreted as causal explanations for the observed group differences.

From a clinical viewpoint, the digital–AI workflow may contribute to shorter chair time, reduced laboratory inventories, and potentially higher patient satisfaction—outcomes echoed by surveys on three-dimensional facial-scan accuracy and AI-driven patient engagement platforms [14,40]. Economic evaluations are underway; however, in-office metal printing already matches commercial turnaround times while allowing for rapid design iterations [17,41]. These efficiencies are particularly attractive for complex malocclusions and geographically distant patients, populations traditionally characterised by extended treatment times and higher dropout rates. Yet, because treatment time and patient-reported outcomes were not collected in this trial, such clinical advantages cannot be inferred from the present findings. Digital workflows may require additional equipment investment, training, and administrative workload, which were not assessed in this trial. Retention appliances were delivered only after outcome assessment; therefore, the retention protocol did not influence the measured PAR results and should not be interpreted as a source of bias between groups.

Despite its strengths—including a prospective design, blinded assessments, strict adherence to CONSORT guidelines, and advanced image registration protocols—the study has limitations. Because retention appliances were delivered only after outcome assessment, they did not influence the PAR-based comparisons and should not be regarded as a source of bias. It was conducted at a single academic centre; thus, operator expertise may partially account for outcomes. Differences in imaging modalities (CBCT versus 2D radiography) may introduce information bias and could have influenced diagnostic precision and treatment planning; therefore, the observed differences should be interpreted as reflecting an integrated digital workflow rather than the isolated effect of AI-assisted planning. Furthermore, retention follow-up in this study was limited to immediate post-treatment records, and no long-term assessment of post-treatment stability was performed, which restricts the interpretation of outcome durability. The 12-month follow-up does not capture long-term stability or root-resorption risk, issues of special relevance for patients with syndromic cranio-facial conditions or down-syndrome-associated periodontal vulnerability [42]. Biomarkers of bone metabolism were not measured; future work employing coarse-to-fine 3-D tooth-segmentation pipelines [43] and salivary assays could elucidate mechanistic pathways. Finally, although logistic-regression techniques appropriate for complex data were applied [35], residual confounding by psychosocial or operator variables cannot be excluded.

Future studies should therefore adopt multicentre designs, incorporate cost-utility analyses and extend observation windows to capture post-retention stability. Integrating hyperspectral bone-turnover imaging with algorithm-guided force systems may reveal how digital mechanics interact with cytokine-mediated remodelling [37]. Parallel advances in additive manufacturing for maxillofacial prosthodontics [19,44,45] suggest that an engineering-biology pipeline will continue to personalise orthodontic intervention.

## 5. Conclusions

Under controlled trial conditions, the AI-assisted digital orthodontic workflow produced statistically significant and clinically interpretable short-term improvements in PAR scores compared with conventional fixed appliances. These benefits likely reflect the combined influence of high-precision imaging, customized appliance fabrication, and AI-supported monitoring, and cannot be attributed to AI alone.

Because outcomes were assessed immediately after treatment and only Class I cases from a single center were included, the durability and generalizability of these findings remain limited. Future multicenter studies with extended post-retention follow-up and cost–utility analyses are required to determine the long-term clinical value of this integrated digital workflow.

## Supporting information

S1 Data S1 Fig. PAR scoring scheme illustrated with standardized line drawings. Schematic illustration of the five components of the Peer Assessment Rating (PAR) index used to evaluate occlusal outcomes. (A) Upper anterior alignment and (B) lower anterior alignment represent crowding or spacing of the maxillary and mandibular anterior segments, respectively. (C) Overjet reflects the anteroposterior relationship of the anterior teeth. (D) Overbite/open bite depicts the vertical relationship of the anterior teeth. (E) Buccal occlusion illustrates posterior occlusal relationships, incorporating anteroposterior, vertical, and transverse dimensions. S2 Fig. Scatter plots of baseline versus post-treatment PAR scores at T2. Each dot represents an individual participant. Regions indicate categories of change (worse/no difference, improved, greatly improved). Most participants in the Digital and AI group clustered within the “greatly improved” region, whereas those in the Conventional group were more widely distributed across the “partial improvement” and “no improvement” categories. S1 File. Study protocol. S2 File. CONSORT 2010 checklist of information to include when reporting a randomised trial.
(ZIP)
