# Supplementary material for: Randomized controlled trial comparing AI-assisted digital and conventional orthodontics: Superior PAR reduction and occlusal outcomes
Source: PLoS One. 2026 May 4;21(5):e0347499. doi: 10.1371/journal.pone.0347499 (PMC13138670; doi:10.1371/journal.pone.0347499)
Supplement: S1 Data — Schematic illustration of the five components of the Peer Assessment Rating (PAR) index used to evaluate occlusal outcomes. (A) Upper anterior alignment and (B) lower anterior alignment represent crowding or spacing of the maxillary and mandibular anterior segments, respectively. (C) Overjet reflects the anteroposterior relationship of the anterior teeth. (D) Overbite/open bite depicts the vertical relationship of the anterior teeth. (E) Buccal occlusion illustrates posterior occlusal relationships, incorporating anteroposterior, vertical, and transverse dimensions. S2 Fig. Scatter plots of baseline versus post-treatment PAR scores at T2. Each dot represents an individual participant. Regions indicate categories of change (worse/no difference, improved, greatly improved). Most participants in the Digital and AI group clustered within the “greatly improved” region, whereas those in the Conventional group were more widely distributed across the “partial improvement” and “no improvement” categories. S1 File. Study protocol. S2 File. CONSORT 2010 checklist of information to include when reporting a randomised trial. (ZIP) [file pone.0347499.s001.zip › 01 S1 Fig.pdf]

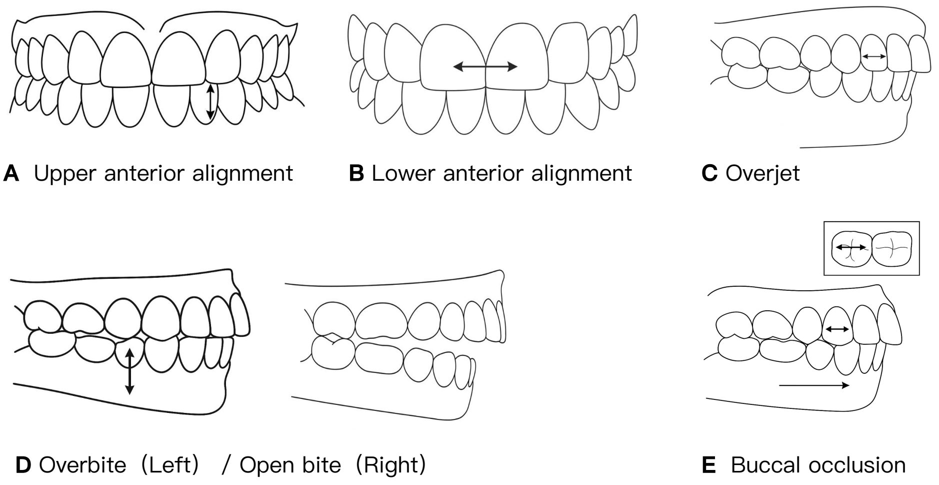

Fig S1. PAR Scoring Scheme Illustrated with Standardized Line Drawings.

Schematic illustration of the five components of the Peer Assessment Rating (PAR) index used to evaluate occlusal outcomes.

(A) Upper anterior alignment and (B) lower anterior alignment represent crowding or spacing of the maxillary and mandibular anterior segments, respectively.

(C) Overjet reflects the anteroposterior relationship of the anterior teeth.

(D) Overbite / open bite depicts the vertical relationship of the anterior teeth.

(E) Buccal occlusion illustrates posterior occlusal relationships, incorporating anteroposterior, vertical, and transverse dimensions.
