# Supplementary material for: Randomized controlled trial comparing AI-assisted digital and conventional orthodontics: Superior PAR reduction and occlusal outcomes
Source: PLoS One. 2026 May 4;21(5):e0347499. doi: 10.1371/journal.pone.0347499 (PMC13138670; doi:10.1371/journal.pone.0347499)
Supplement: S1 Data — Schematic illustration of the five components of the Peer Assessment Rating (PAR) index used to evaluate occlusal outcomes. (A) Upper anterior alignment and (B) lower anterior alignment represent crowding or spacing of the maxillary and mandibular anterior segments, respectively. (C) Overjet reflects the anteroposterior relationship of the anterior teeth. (D) Overbite/open bite depicts the vertical relationship of the anterior teeth. (E) Buccal occlusion illustrates posterior occlusal relationships, incorporating anteroposterior, vertical, and transverse dimensions. S2 Fig. Scatter plots of baseline versus post-treatment PAR scores at T2. Each dot represents an individual participant. Regions indicate categories of change (worse/no difference, improved, greatly improved). Most participants in the Digital and AI group clustered within the “greatly improved” region, whereas those in the Conventional group were more widely distributed across the “partial improvement” and “no improvement” categories. S1 File. Study protocol. S2 File. CONSORT 2010 checklist of information to include when reporting a randomised trial. (ZIP) [file pone.0347499.s001.zip › 03 S1 File‎.pdf]

研究方案

项目名称：数字化与人工智能辅助正畸治疗临床研究：一项随机对照试验

方案版本号及日期：（V1.0，2023 年 7 月 2 日）

研究起止时间：（2023 年 9 月-2025 年 9 月）

一、研究背景

错颌畸形是口腔常见疾病，严重影响患者的咀嚼功能、颌面美观及口腔健康相关生活质量。传统固定矫治（托槽-弓丝系统）虽然已经形成了较为成熟的诊疗体系，但在诊断分析、治疗设计及临床操作过程中仍然高度依赖医生的主观判断。由于医师经验差异、操作习惯及患者依从性等因素影响，治疗效果存在一定的差异性，尤其在咬合收尾阶段，可能导致疗效和效率的不一致。

近年来，随着数字化口腔医学的快速发展，高精度口内扫描、锥形束 CT（CBCT）及虚拟三维建模技术被引入口腔正畸领域，可显著提高诊断与治疗设计的客观性与可重复性。同时，三维打印技术能够实现个性化矫治器械的快速制作，减少人工误差。与此同时，人工智能（AI）算法的引入，使得牙颌标志点识别、牙齿移动预测及远程监测成为可能，为治疗过程的自动化与精细化提供了技术支持。

然而，现有关于数字化与 AI 辅助正畸的研究多为实验室研究、回顾性分析或病例系列，缺乏严格的前瞻性随机对照试验。尤其在以 Peer Assessment Rating（PAR）指数 这一国际公认的客观评价工具来衡量矫治效果的研究方面，证据仍显不足。因此，需要通过高质量的临床随机对照试验来评价数字化与 AI 辅助正畸在咬合质量改善方面的真实临床价值。

二、研究目的

本研究旨在比较 人工智能辅助数字化正畸工作流 与 传统固定矫治 在改善咬合质量方面的效果差异。

- 主要目的：评估两种矫治方式在基线（T0）至治疗完成（T2）期间 PAR 改善率（%） 的差异。
- 次要目的：比较两组在治疗完成时的 总 PAR 值、达到 ≥70% PAR 改善 的比例，以及 PAR 各组成部分（上/下前牙排齐、覆/开合、颊侧关系及中线）的变化情况。

研究假设：与传统固定矫治相比，数字化与 AI 辅助正畸能够实现更高的 PAR 改善率，并在终末咬合质量上具有优势。

三、立题依据

1. 研究必要性

- 传统正畸流程存在主观性强、疗效差异大等问题，需

要新技术来提升诊疗的客观性与一致性。

- 数字化与 AI 技术已在正畸中逐步应用，但缺乏高质量随机对照试验证据。
- PAR 指数作为国际通用的咬合评价工具，能够全面反映治疗前后的咬合改善情况，适合作为客观终点指标。

2. 研究可行性

- 本研究团队具备数字化口腔设备（TRIOS 口扫、CBCT、3Shape 软件、Form 3B+ 打印机）及 AI 远程监测平台（DentalMonitoring™），并拥有完整的正畸诊疗与随访体系。
- 研究人员具备丰富的临床经验，并已建立标准化的评估者训练流程，确保 PAR 评分的一致性（ICC≥0.90）。

3. 研究价值

- 本研究结果可直接回答数字化与 AI 工作流是否能够在临床实践中实现 更高质量的咬合改善。
- 若假设成立，将为数字化与 AI 在正畸中的推广应用提供循证依据。
- 同时，本研究的设计与方法学（RCT、盲法评估、预注册及统计计划）可为后续多中心研究及卫生经济学评价提供参考。

四、研究内容

1. 研究对象

本研究拟纳入在川北医学院附属医院口腔科正畸门诊就诊的患者。

- 纳入标准：年龄 12–35 岁；依据 BSI 标准确诊为安氏 I 类错颌畸形；无影响骨代谢的全身疾病；既往无正畸治疗史；能够按时复诊并完成随访。
- 排除标准：妊娠或哺乳期女性；活动性牙周疾病；严重心理或行为障碍；主治医生判定的其他不适宜参与本研究的情况。

2. 研究设计与方法

- 研究类型：前瞻性、单中心、平行分组、随机对照试验。
- 分组方法:按照 1:1 比例将受试者分配至 数字-AI 组与 传统组。随机序列由独立统计人员使用 R 软件生成（区组大小 4),分配隐藏通过连续编号、密封不透明信封(SNOSE)实现。
- 干预措施：
- 数字-AI 组:口内扫描(TRIOS 4)、CBCT(iCAT FLX V10)、3Shape 软件虚拟设计，Form 3B+ 3D 打印托槽及附件，DentalMonitoring™ 远程监测；
- 传统组：藻酸盐印模、全景片和头颅侧位片，MBT 处

方直丝弓托槽，常规 4 周复诊激活。

- 流程标准化：两组均由同一组具备 ≥5 年临床经验的正畸专科医师完成操作，复诊频率、保持方案一致，确保除干预方式外其他环节可比。

3. 观察指标

- 主要结局指标：
- PAR 改善率（%）：[(T0 PAR – T2 PAR) / T0 PAR] × 100%，T2 为治疗完成时点。
- 次要结局指标：
- 治疗完成时的总 PAR 值；
- 治疗完成时达到 ≥70% PAR 改善 的比例；
- 不同时间点（T0、T1–6m、T1–12m、T2）各 PAR 组成部分的变化（前牙排齐、覆/开合、颊侧关系、中线）。
- 评估方法：由 3 名经校准训练的正畸医师独立完成评分，盲法判读，计算 ICC 评估一致性，目标 ICC ≥0.90。

4. 样本量估算

基于双侧独立样本 t 检验，设定 α=0.05、检验效能（1-β）=0.80，中等效应量（Cohen’s d=0.5），计算每组需要 63 例。考虑约 10% 脱落，最终确定每组 70 例，总样本量 140 例。

5. 统计学方法

• 主要结局（T2 时 PAR 改善率）：组间比较采用独立样本 t 检验，报告均值差（MD）及 95% CI。

• 次要结局：

• 总 PAR 值（t 检验）；

• ≥70% 改善比例（χ^2 或 Fisher 精确检验，计算 RR 及 95% CI）；

• 各时间点纵向变化（重复测量方差分析，必要时进行 Greenhouse–Geisser 校正）。

• 缺失数据：若比例 <5%，采用多重插补法（m=5，预测均值匹配）；分析集包括 ITT 和 PP。

• 软件：SPSS 29.0 和 R 4.2.2。

6. 受试者权益与风险控制

本研究涉及的受试者权益保护与风险—受益评估详见第五部分。

五、研究对象权益保护与风险—受益评估

1. 研究对象权益保护

a. 知情同意

• 在入组前，由研究人员向所有潜在受试者及其监护人

（如为未成年人）充分告知研究目的、研究内容、可能风险与受益、受试者权利及退出机制。

• 知情同意采用书面形式，受试者自愿签署后方可正式入组。未成年人需由其法定监护人签署，并在可能的情况下征求未成年人的意见。

• 知情同意书中将明确说明：参与或退出不会影响常规医疗服务质量。

b. 隐私与保密

• 所有研究数据将在收集后去标识化处理，每位受试者赋予唯一编码。

• 影像学资料与随访数据存储于 AES-256 加密数据库，仅研究团队指定人员可访问。

• 在学术交流与论文发表中，仅呈现汇总结果，不会披露受试者个人身份信息。

c. 自愿与退出

• 参与研究完全自愿，受试者有权在任何时间无条件退出研究，退出不会对后续治疗造成任何不良影响。

• 退出前已采集的数据仍会用于 ITT 分析，以确保研究结果的完整性。

d. 补偿与保障

• 本研究不收取任何额外费用，所有诊疗费用按医院常规收取。

- 若因研究导致意外损伤，将由研究团队和医院按照相关规定负责处理。
- 研究方案属最小风险范畴，不设专门保险。

2. 风险—受益评估

a. 可能风险

- 常规正畸风险：如托槽脱落、弓丝刺激、口腔软组织轻度不适，均可通过常规处理解决。
- 影像学风险：数字-AI 组在诊断阶段需进行 CBCT 检查，存在低剂量辐射风险；将严格遵循 ALARA（As Low As Reasonably Achievable） 原则，仅在必要时获取 CBCT。
- 数据隐私风险：远程监测涉及图像上传与云端处理，可能存在信息泄露风险；本研究采取数据加密与最小必要原则，确保安全。

b. 风险最小化措施

- 研究人员具备丰富临床经验，可及时处理常见不良事件。
- 使用符合国家标准的 CBCT 设备，并由影像科及正畸科医师共同把关检查指征。
- 数据存储采用加密服务器，访问需授权，严格控制数据外泄风险。

c. 潜在受益

- 直接受益：受试者有望通过数字-AI 流程获得更高精度的诊疗设计与更佳的咬合结局，同时提高依从性。
- 间接受益：研究结果将为未来正畸流程优化、临床指南更新及患者获益提供循证依据。

d. 风险—受益平衡

- 本研究的所有风险均属于常规临床诊疗中可能出现的情形，额外风险极小。
- 受试者有机会从先进的数字化与 AI 技术中直接受益，同时研究结论将推动正畸学科发展。
- 综上，本研究属于 最小风险研究，其潜在受益明显大于潜在风险。

六、研究设计、技术路线、实验手段与研究流程

1. 总体研究设计

本研究为单中心、前瞻性、随机对照试验。受试者按 1:1 比例分配至数字-AI 组与传统组，比较两组从基线（T0）至完成时（T2）的 PAR 改善情况。研究遵循 CONSORT 规范，强调分配隐藏、盲法评估及 ITT 分析，以保证研究的科学性与结果的可靠性。

2. 技术路线

研究整体流程分为五个阶段：

1. 伦理审批与注册 → 完成 IRB 审批及 ChiCTR 注册；

2. 受试者招募与入组 → 按标准筛查，签署知情同意；

3. 随机分组与干预实施 → 使用区组随机和 SNOSE 隐藏，分别接受数字-AI 与传统矫治；

4. 随访与数据采集 → 在 T0、T1–6m、T1–12m、T2 收集模型及影像学数据；

5. 结局评估与统计分析 → 盲法评估者完成 PAR 评分，数据分析遵循预设统计方案。

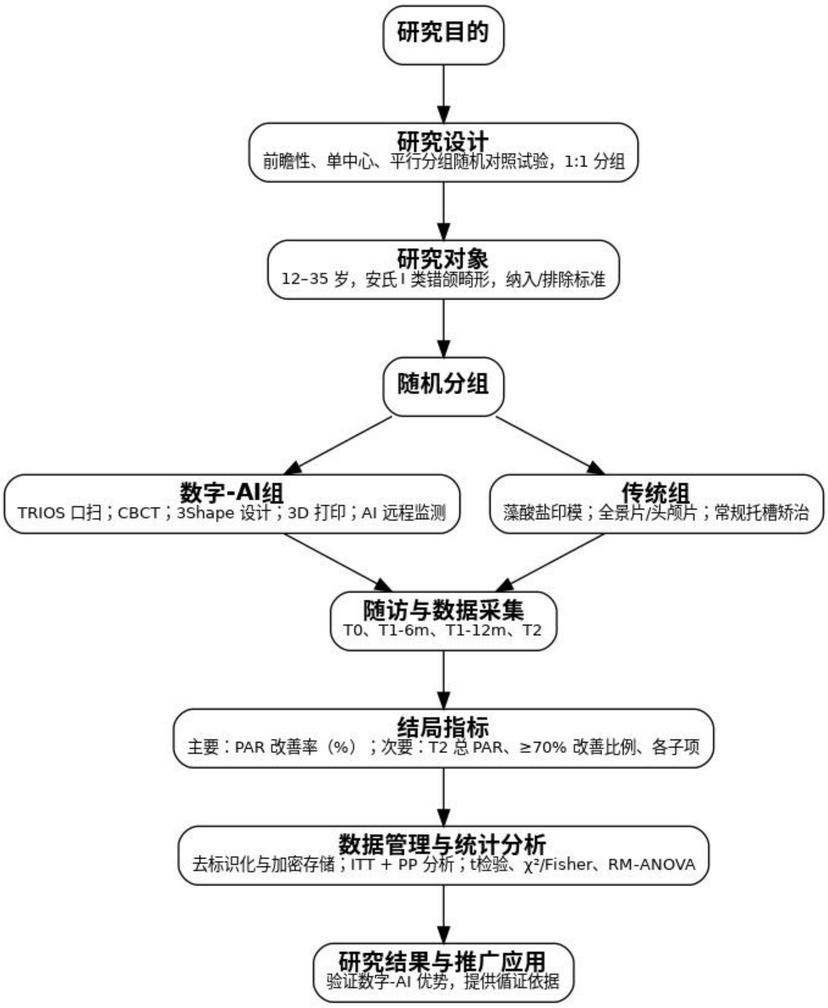

3. 实验手段与工具

- 数字化与 AI 技术：TRIOS 口内扫描、iCAT FLX V10 CBCT、3Shape 设计、Form 3B+ 3D 打印、DentalMonitoring™ 远程监测；
- 结局评估工具：BSI 修订版 PAR 指数评分表，由校准后的评估者完成盲法测量；
- 统计学工具：SPSS 29.0 与 R 4.2.2，用于数据录入与分析。

4. 研究流程示意

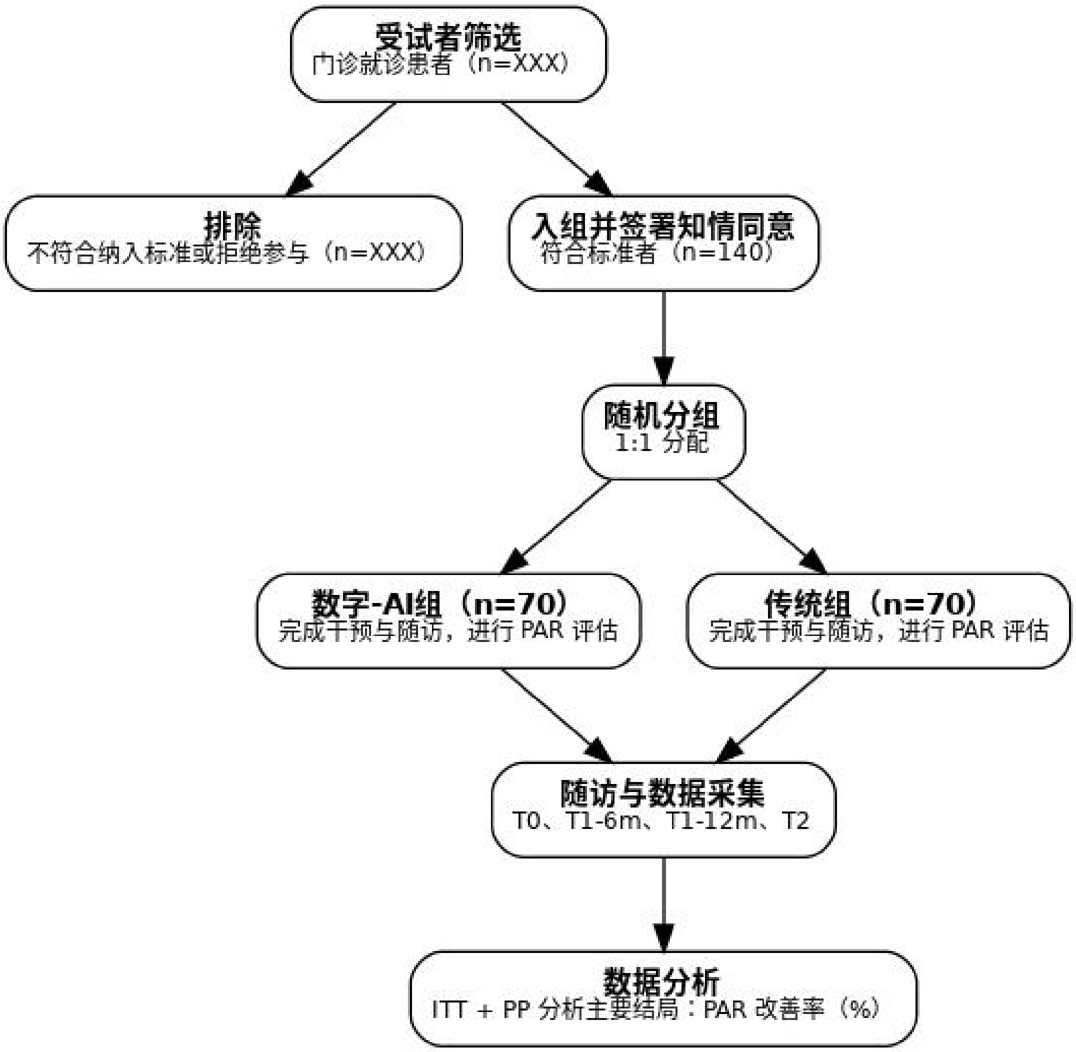

七、知 情 同 意

知 情 同 意 书（模板）

尊敬的受试者：

您好！我们诚挚邀请您参加由川北医学院附属医院口腔科开展的一项临床研究。本研究题目为 “数字化与人工智能辅助正畸治疗对缩短治疗时间的临床研究：一项随机对照试验”。研究项目已通过川北医学院附属医院伦理委员会审查(批准号：****）。请您在决定是否参加之前，仔细阅读以下内容。

1. 研究背景

正畸治疗通常需要较长的时间，可能增加患者不适和并发症风险。随着数字化和人工智能技术的发展，新型工作流程有望缩短治疗时间、提升效率，但仍缺乏高质量临床证据。因此，我们计划开展随机对照试验，对比 数字化与人工智能辅助的正畸治疗 与 传统正畸治疗 在治疗时间及相关效果上的差异。

2. 研究目的

本研究旨在比较数字化与人工智能辅助正畸治疗与传统正畸治疗在 总治疗时间 方面的差异，并评估其在诊断、治疗实施、保持阶段的效率和安全性。

3. 研究内容和步骤

本研究为单中心随机对照试验，共计划招募 140 名受试者（每组 70 人）。

入选条件：年龄 12–35 岁，Angle I 类错颌畸形，无严重全身疾病或既往正畸史。

排除条件：妊娠或哺乳期、严重牙周病、颅颌面畸形、需手术者等。

分组方式：通过计算机区组随机分配（1:1 比例），受试者将有相等的机会进入 数字化与 AI 组 或 传统组。

治疗方案：

数字化与 AI 组：采用口内扫描、CBCT 影像、AI 分析、CAD/CAM 和 3D 打印托槽保持器、AI 随访。

传统组：采用常规印模和石膏模型、二维影像与人工诊断、传统托槽弓丝、实验室保持器、常规随访。

治疗期间：需按时复诊，接受规范操作；部分病例可能采集口腔影像和记录，用于研究分析。

4. 研究中需要您哪些方面的配合

按照医生安排完成所有治疗和复诊；

在研究期间配合影像学检查（如 CBCT、X 片、口内扫描）；

按时填写或接受满意度调查问卷。

5. 参加研究可能的风险、不适及处理方法

治疗风险：包括常见的正畸风险（牙根吸收、釉质脱矿、牙龈炎症、牙齿轻度不适等）；

影像学检查：CBCT 属于低剂量 X 射线检查，存在少量辐射风险，但在安全范围内；

数字化与 AI 系统尚属新技术，存在轻微操作延迟或设备故障的可能。

一旦出现不良事件,研究团队将及时处理并提供必要的医疗支持。相关治疗费用由研究团队或医院承担。

6. 参加研究可能的受益与补偿

您可能从数字化和人工智能带来的更短治疗时间、更少复诊次数中获益；

您的治疗方案与常规正畸治疗相同，均符合临床标准，不会因此降低治疗效果；

研究结果将有助于提高未来正畸治疗效率和质量；

如有额外检查费用，由研究项目承担；复诊交通费可根据情况补偿。

7. 自愿参加/退出研究

参加本研究以自愿为原则。您可以拒绝参加或随时退出研究，您不会因此而受到歧视、不公正对待或报复，您的医疗待遇与权益不会受到任何影响。

8. 个人信息的保密

在研究期间收集到的所有信息都将是保密的,并由研究者保管。 研究人员、伦理委员会成员及相关管理部门在法律允许的范围内，有权审阅您的信息记录。在任何有关本项目的研究报告和出版物中，您的个人信息不会被独立公开。

9. 联系方式

您可以在任何时间提出有关本项研究的任何问题。 您可以与您的医生取得联系，联系人：谢小亭 ，联系电话： 13458223809。如果您对参加项目有任何的抱怨，请联系川北医学院伦理委员会 （联系电话 0817-3371779）。

同 意 声 明

1 、我已认真阅读了上述有关本研究的介绍， 而且有机会就此研究与医生讨论并提出问题。我提出的所有问题都得到了满意的答复。

2 、我知道参加研究是自愿的， 我确认已有充足的时间对此进行考虑，而且明白：

（1）我已了解参加该试验可能发生的潜在风险及风险发生后的治疗。

（2）我已了解本试验的相关替代治疗方案。

（3）我可以随时向医生咨询更多的信息。

（4）我可以随时退出研究， 而不受到歧视和报复， 医疗待遇与权益不会受到影响。

最后，我决定同意参加本项研究，并愿意按研究方案要求与医生配合完成本研究。

受试者签字： 日期： 电话：

监护人签字： 日期： 电话：

我已向该受试者充分解释和说明了本项研究的目的、操作过程以及受试者参加该项目可能存在的风险和利益，并满意地回答了受试者的所有有关问题。

研究者签名： 日期： 电话：

八、参与研究者

临床试验参加研究者一览表

姓名	性别	职称	学历	专业	是否经过GCP培训	研究分工	签名
刘英	女	教授	博士	口腔	是	研究负责人，总体学术与行政负责，方案把关，进度/质量/经费统筹	刘英
谢小亭	女	讲师	博士	口腔	是	临床总协调，门诊流程统筹，复杂病例会诊，临床质量控制	谢小亭
伍建荣	男	主管护师	研究生	口腔	是	随机化与统计学负责人，区组随机序列生成，统计方案审核	伍建荣
伏燕	女	主管护师	研究生	口腔	是	分配隐藏与信封管理，入组分配登记，稽核监督	伏燕
米燕香	女	主管护师	研究生	口腔	是	受试者招募与筛查协调，建立招募台账，初筛与报表	米燕香
刘思	女	主管护师	研究生	口腔	是	知情同意与伦理联络，文件管理，批件递交，投诉处理	刘思
李娜	女	副教授	博士	口腔	是	基线评估与口腔常规检查，病史采集，CRF 填写	李娜
琼仙	女	讲师	博士	影像	是	影像学负责人，CBCT 与二维影像采集、质控与备份	琼仙
龚宇红	女	护师	本科	口腔	是	数字化扫描与模型工程，口扫，STL 管理，质控	龚宇红
吴曾波	男	副教授	博士	口腔	是	AI 分析与治疗计划，辅助测量与模拟，输出计划版本	吴曾波
何欢	女	护师	本科	口腔	是	CAD/CAM 与 3D 打印主管，保持器设计、制作与交付台账	何欢

陈定根	男	副教授	硕士	口腔	是	数字化与 AI 组临床负责人，实验组治疗实施与随访	[illegible]
晏燕	女	副主任医师	硕士	口腔	是	传统组临床负责人，对照组常规治疗与记录	晏燕
刘华	女	讲师	硕士	口腔	是	盲法结局测量负责人，主要/次要结局独立测量与复核	刘华
王伟丽	女	讲师	硕士	口腔	是	盲法结局评估员 A，结局数据核对与录入	王伟丽
郭黎	女	讲师	硕士	口腔	是	盲法结局评估员 B，交叉复核与一致性报告	郭黎
朱万春	男	副教授	博士	口腔	是	数据管理负责人，CRF 设计、双录入、数据清理	朱万春
师敏	女	讲师	博士	口腔	是	生物统计与分析，执行 SAP，回归与多重校正	师敏
黄豆	男	护师	本科	口腔	是	不良事件/安全官，AE/SAE 判定与上报，安全报告	黄豆
青松	男	副教授	博士	口腔	是	质量控制与监查，SOP 依从性稽核，CAPA 整改	[illegible]

Study Protocol

Project Title: Clinical Study of Digital and Artificial Intelligence-Assisted Orthodontic Treatment: A Randomized Controlled Trial
Protocol Version and Date: (V1.0, July 2, 2023)
Study Period: (September 2023 – September 2025)

I. Background

Malocclusion is a common oral condition that seriously affects patients' masticatory function, facial aesthetics, and oral health-related quality of life. Although conventional fixed appliance therapy (bracket–archwire system) has developed into a relatively mature treatment modality, the processes of diagnosis, treatment planning, and clinical operation still heavily rely on clinicians' subjective judgment. Due to differences in clinician experience, operational habits, and patient compliance, treatment outcomes show considerable variability. This variability is particularly evident in the finishing stage, which may lead to inconsistencies in treatment efficacy and efficiency.

In recent years, with the rapid development of digital dentistry, high-precision intraoral scanning, cone-beam computed tomography (CBCT), and virtual three-dimensional modeling technologies have been introduced into orthodontics, significantly improving the objectivity and reproducibility of diagnosis and treatment planning. At the same time, three-dimensional printing technology enables the rapid fabrication of personalized orthodontic appliances, reducing human error. Moreover, the introduction of artificial intelligence (AI) algorithms has made possible the recognition of dental landmarks, prediction of tooth movement, and remote monitoring, providing technical support for automation and refinement of the treatment process.

However, most existing studies on digital and AI-assisted orthodontics are laboratory-based, retrospective analyses, or case series, and lack rigorous prospective randomized controlled trials. In particular, studies that employ the Peer Assessment Rating (PAR) index, an internationally recognized objective tool for evaluating orthodontic outcomes, are still insufficient. Therefore, high-quality clinical randomized controlled trials are needed to evaluate the true clinical value of digital and AI-assisted orthodontics in improving occlusal quality.

II. Objectives

This study aims to compare the effectiveness of digital and AI-assisted orthodontic workflow and conventional fixed appliance therapy in improving occlusal quality.

Primary Objective: To evaluate the difference in PAR improvement rate (%) between baseline (T0) and treatment completion (T2) in the two groups.

Secondary Objectives: To compare the total PAR score at T2, the proportion of participants achieving ≥70% PAR improvement, and changes in the PAR components (upper/lower anterior alignment, overjet/open bite, buccal occlusion, and midline) between the two groups.

Hypothesis: Compared with conventional fixed appliance therapy, digital and AI-assisted orthodontics can achieve a higher PAR improvement rate and demonstrate advantages in final occlusal quality.

III. Rationale

Necessity of the Study

Conventional orthodontic procedures are highly subjective, and treatment outcomes vary considerably; new technologies are needed to improve objectivity and consistency.

Although digital and AI technologies have been increasingly applied in orthodontics, high-quality randomized controlled trial evidence is still lacking.

The PAR index, as an internationally accepted occlusal evaluation tool, can comprehensively reflect pre- and post-treatment changes and is suitable as an objective endpoint.

Feasibility

The study team is equipped with advanced digital dental equipment (TRIOS intraoral scanner, CBCT, 3Shape software, Form 3B+ 3D printer) and an AI-based remote monitoring platform (DentalMonitoring™), as well as a complete orthodontic treatment and follow-up system.

Investigators have extensive clinical experience and have established standardized training for evaluators to ensure consistency in PAR scoring (ICC ≥0.90).

Value of the Study

The results of this study can directly answer whether digital and AI-assisted workflows can achieve higher quality occlusal improvements in clinical practice.

If the hypothesis is confirmed, the study will provide evidence to support the broader application of digital and AI technologies in orthodontics.

In addition, the study design and methodology (RCT, blinded assessment, preregistration, and statistical plan) can serve as a methodological reference for subsequent multicenter studies and health economic evaluations.

IV. Study Content

1. Participants

This study plans to include patients attending the orthodontic clinic of the Department of Stomatology, Affiliated Hospital of North Sichuan Medical College.

Inclusion criteria: Aged 12–35 years; diagnosed as Angle Class I malocclusion according to BSI standards; free of systemic diseases affecting bone metabolism; no prior orthodontic treatment history; able to attend scheduled visits and complete follow-up.

Exclusion criteria: Pregnant or lactating women; active periodontal disease; severe psychological or behavioral disorders; any other conditions judged by the attending orthodontist as unsuitable for participation.

2. Study Design and Methods

Study type: Prospective, single-center, parallel-group, randomized controlled trial.

Grouping method: Participants will be allocated 1:1 into the Digital–AI group and the Conventional group. The randomization sequence will be generated by an independent statistician using R software (block size = 4). Allocation concealment will be implemented using sequentially numbered, opaque, sealed envelopes (SNOSE).

Interventions:

Digital–AI group: Intraoral scanning (TRIOS 4), CBCT (iCAT FLX V10), 3Shape software virtual design, customized brackets and auxiliaries fabricated with Form 3B+ 3D printer, and remote monitoring via DentalMonitoring™.

Conventional group: Alginate impression, panoramic and lateral cephalometric

radiographs, MBT prescription straight-wire brackets, routine 4-week adjustment appointments.

Standardization of procedures: All treatments will be performed by the same team of orthodontic specialists with ≥5 years of experience. Visit intervals and retention regimens are standardized across both groups to ensure comparability except for the intervention method.

3. Outcome Measures

Primary outcome measure:

PAR improvement rate (%): [(T0 PAR – T2 PAR) / T0 PAR] × 100%, with T2 defined as treatment completion.

Secondary outcome measures:

Total PAR score at T2;

Proportion achieving ≥70% PAR reduction at T2;

Changes in PAR components (anterior alignment, overjet/open bite, buccal occlusion, and midline) at T0, T1-6m, T1-12m, and T2.

Assessment method: PAR scoring will be performed independently by three calibrated orthodontists under blinded conditions. Inter-rater reliability will be evaluated using intraclass correlation coefficients (ICC), with a target ICC ≥0.90.

4. Sample Size Estimation

Based on a two-sided independent-samples t-test with $\alpha=0.05$, power $(1-\beta)=0.80$, and a medium effect size (Cohen’s d=0.5), 63 participants are required per group. Considering an estimated 10% dropout rate, the final sample size is set at 70 participants per group, for a total of 140 participants.

5. Statistical Methods

Primary outcome (PAR improvement rate at T2): Between-group comparison will be conducted using independent-samples t-test, reporting mean difference (MD) and 95% confidence intervals (CI).

Secondary outcomes:

Total PAR score at T2 (t-test);

Proportion achieving ≥70% improvement (χ^2 test or Fisher's exact test, reporting relative risk [RR] and 95% CI);

Longitudinal changes across timepoints (repeated-measures ANOVA, with Greenhouse–Geisser correction when necessary).

Handling of missing data: If missing data <5%, multiple imputation (m=5, predictive mean matching) will be performed. Analyses will include both ITT and PP sets.

Software: SPSS 29.0 and R 4.2.2.

6. Protection of Participants' Rights and Risk Control

The protection of participants' rights and the risk–benefit evaluation are detailed in Section V.

V. Protection of Participants' Rights and Risk–Benefit Evaluation

1. Protection of Participants' Rights

a. Informed Consent

Before enrollment, investigators will fully inform all potential participants and their guardians (for minors) about the study objectives, procedures, possible risks and benefits, participants' rights, and withdrawal mechanism.

Written informed consent will be obtained before enrollment. For minors, consent will be signed by their legal guardians, and assent will be sought from minors whenever feasible.

The consent form will clearly state that participation or withdrawal will not affect the quality of routine medical care.

b. Privacy and Confidentiality

All study data will be de-identified after collection, with each participant assigned a unique code.

Radiographic and follow-up data will be stored in an AES-256 encrypted database, accessible only to designated members of the research team.

In academic presentations and publications, only aggregated results will be reported, and no individual-identifiable information will be disclosed.

c. Voluntary Participation and Withdrawal

Participation in this study is entirely voluntary. Participants may withdraw from the study at any time without providing a reason, and withdrawal will not affect their subsequent medical care.

Data collected prior to withdrawal will be included in ITT analysis to ensure completeness of the study results.

d. Compensation and Safeguards

This study will not incur any additional costs for participants, and all treatment fees will follow hospital routine charges.

If any unexpected harm occurs due to the study, the research team and hospital will provide appropriate management in accordance with relevant regulations.

As this study is classified as minimal risk, no special insurance is arranged.

2. Risk–Benefit Evaluation

a. Potential Risks

Routine orthodontic risks: Bracket debonding, archwire irritation, or mild soft tissue discomfort, which can be resolved by standard clinical management.

Imaging risks: Participants in the Digital–AI group will undergo CBCT during diagnosis, with a risk of low-dose radiation. The ALARA principle (As Low As Reasonably Achievable) will be strictly followed, and CBCT will only be performed when clinically necessary.

Data privacy risks: Remote monitoring involves image uploading and cloud-based processing, which may carry a risk of data leakage. Data encryption and the principle of minimum necessity will be applied to ensure security.

b. Risk Minimization Measures

The investigators are experienced orthodontists capable of promptly handling common adverse events.

CBCT equipment meeting national standards will be used, and all imaging indications will be jointly reviewed by radiology and orthodontic specialists.

Data will be stored on encrypted servers with controlled access to minimize the risk of leakage.

c. Potential Benefits

Direct benefits: Participants may benefit from more precise treatment planning and improved occlusal outcomes under the Digital–AI workflow, as well as enhanced compliance.

Indirect benefits: The study findings may contribute to optimizing orthodontic workflows, updating clinical guidelines, and improving patient care in the future.

d. Risk–Benefit Balance

All risks involved in this study are consistent with those encountered in routine orthodontic treatment, with minimal additional risk.

Participants may directly benefit from advanced digital and AI-assisted technologies, and the study results will promote the development of orthodontics.

Overall, this study is classified as minimal risk research, with potential benefits outweighing potential risks.

VI. Study Design, Technical Route, Experimental Methods, and Research Flow
1. Overall Study Design

This study is a single-center, prospective, randomized controlled trial. Participants will be randomized 1:1 to the Digital–AI group and the Conventional group, comparing the PAR improvement from baseline (T0) to treatment completion (T2). The study follows CONSORT guidelines, emphasizing allocation concealment, blinded outcome assessment, and ITT analysis to ensure scientific rigor and reliability.

2. Technical Route

The study process consists of five stages:

Ethical approval and registration → IRB approval and ChiCTR registration completed;

Recruitment and enrollment → Screening according to inclusion/exclusion criteria and obtaining written informed consent;

Randomization and intervention → Block randomization with SNOSE concealment,

followed by Digital–AI or conventional orthodontic treatment;

Follow-up and data collection → Models and radiographs collected at T0, T1-6m, T1-12m, and T2;

Outcome evaluation and analysis → PAR scoring performed by blinded evaluators, data analyzed according to the predefined statistical plan.

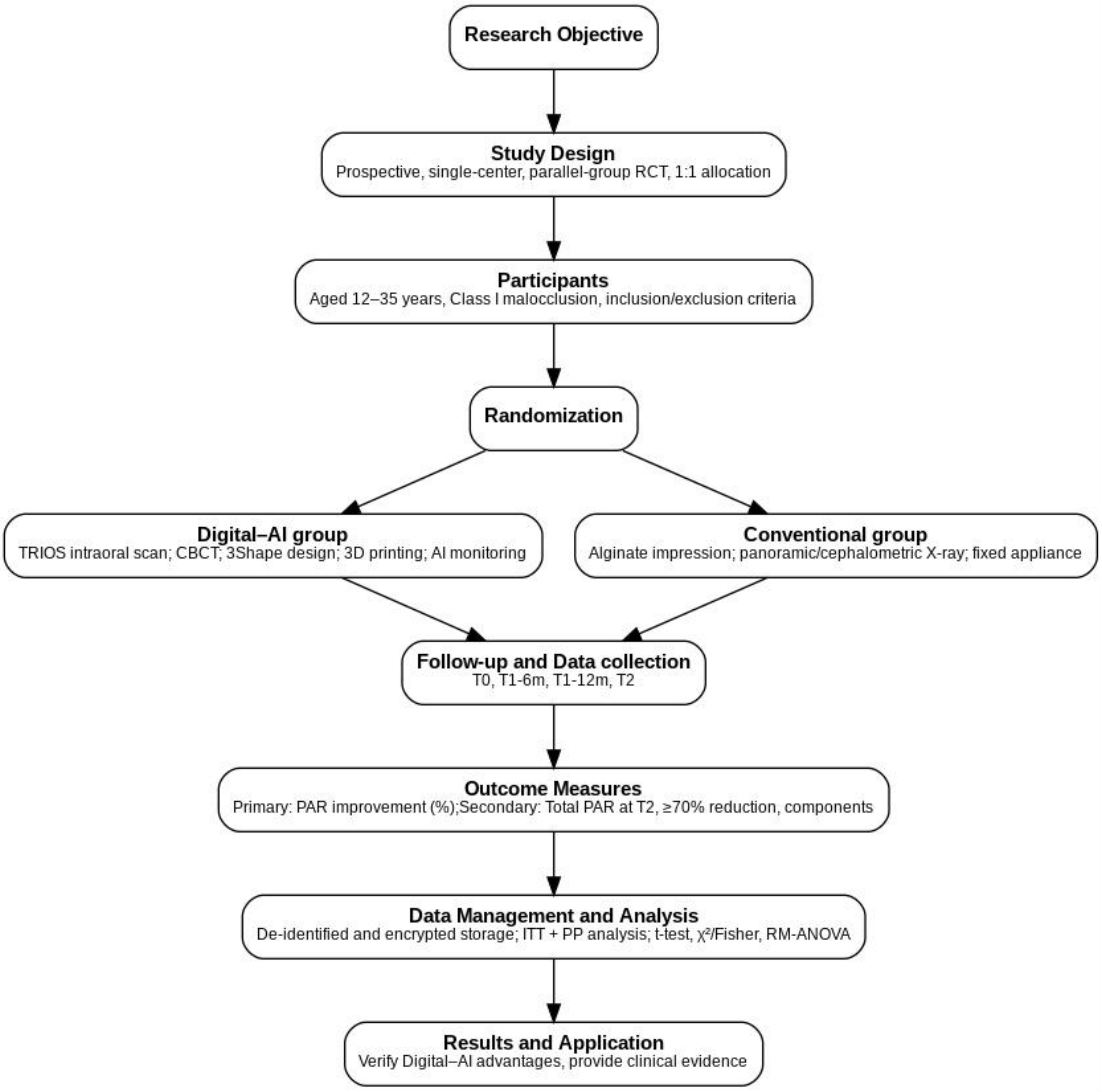

3. Experimental Methods and Tools

Digital and AI technologies: TRIOS intraoral scanner, iCAT FLX V10 CBCT, 3Shape design software, Form 3B+ 3D printer, and DentalMonitoring™ remote monitoring system;

Outcome assessment tool: BSI revised PAR index scoring sheet, with blinded assessments performed by calibrated evaluators;

Statistical tools: SPSS 29.0 and R 4.2.2, used for data entry and statistical analysis.

4. Research Flow Illustration

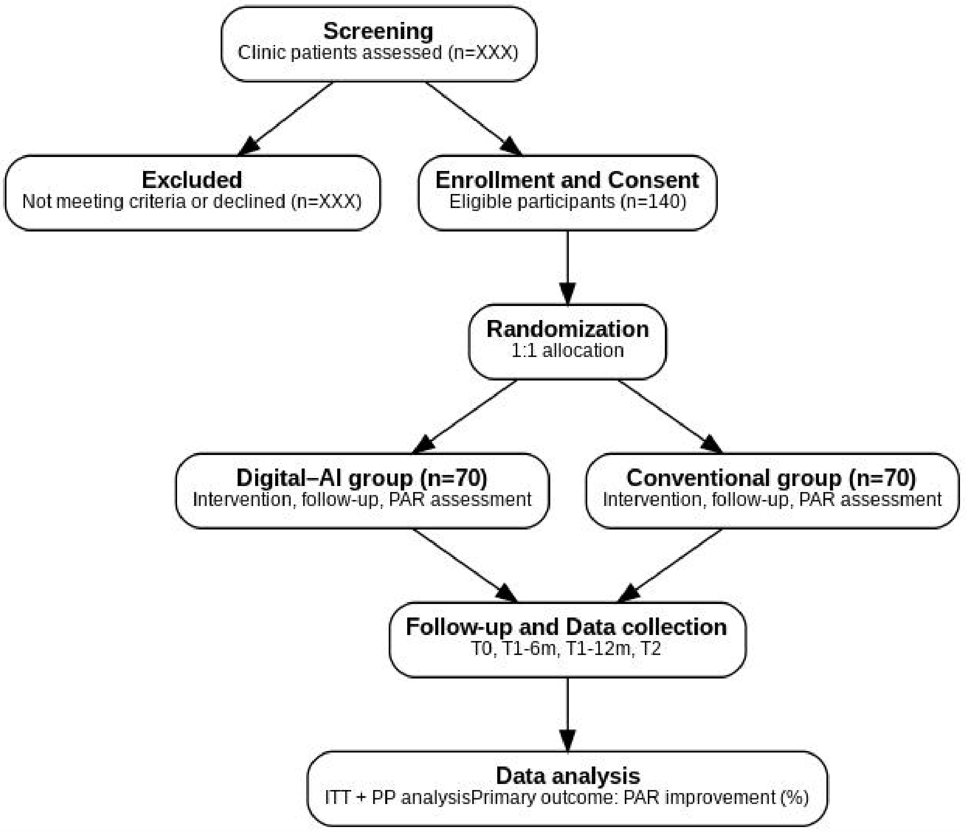

VII. Informed Consent

Informed Consent Form (Template)

Dear Participant:

Greetings! We sincerely invite you to participate in a clinical study conducted by the Department of Stomatology, Affiliated Hospital of North Sichuan Medical College. The title of this study is "Clinical Study of Digital and Artificial Intelligence-Assisted Orthodontic Treatment for Shortening Treatment Duration: A Randomized Controlled Trial." This project has been reviewed and approved by the Ethics Committee of the Affiliated Hospital of North Sichuan Medical College (Approval No.: ****). Before you decide whether to participate, please carefully read the following information.

I. Background

Orthodontic treatment usually requires a long duration, which may increase patient discomfort and the risk of complications. With the development of digital and artificial intelligence (AI) technologies, new workflows may shorten treatment time and improve efficiency; however, high-quality clinical evidence is still lacking. Therefore, we plan to conduct a randomized controlled trial comparing digital and AI-assisted orthodontic treatment with conventional orthodontic treatment in terms of treatment duration and related outcomes.

II. Purpose of the Study

The purpose of this study is to compare the differences in total treatment time between digital and AI-assisted orthodontic treatment and conventional orthodontic treatment, and to evaluate their efficiency and safety in diagnosis, treatment implementation, and retention stages.

III. Study Content and Procedures

This is a single-center randomized controlled trial, with a planned total enrollment of 140 participants (70 per group).

Inclusion criteria: Aged 12–35 years; diagnosed with Angle Class I malocclusion; no severe systemic diseases; no prior orthodontic history.

Exclusion criteria: Pregnant or lactating women; severe periodontal disease; craniofacial deformities; patients requiring surgery, etc.

Grouping method: Participants will be allocated 1:1 by computer-generated block

randomization, with equal probability of entering the Digital–AI group or the Conventional group.

Treatment plans:

Digital–AI group: Intraoral scanning, CBCT imaging, AI analysis, CAD/CAM and 3D printing of brackets and retainers, AI-assisted follow-up.

Conventional group: Conventional impressions and plaster models, 2D radiographs and manual diagnosis, traditional bracket–archwire system, laboratory-fabricated retainers, routine follow-up.

Treatment process: Participants are required to attend scheduled visits and receive standardized treatment. In some cases, oral images and records may be collected for research analysis.

IV. What We Need You to Do

Complete all treatment and follow-up appointments as arranged by the clinician;

Undergo imaging examinations during the study (e.g., CBCT, X-rays, intraoral scanning);

Complete or participate in satisfaction questionnaires during the study.

V. Potential Risks, Discomforts, and Management

Treatment risks: Common orthodontic risks such as root resorption, enamel demineralization, gingival inflammation, or mild dental discomfort;

Imaging risks: CBCT is a low-dose X-ray examination and involves a small amount of radiation, but within safe limits;

Technology risks: Digital and AI systems are relatively new and may occasionally have minor operational delays or equipment failures.

In case of any adverse events, the research team will promptly manage and provide necessary medical support. Any related treatment costs will be covered by the research team or the hospital.

VI. Potential Benefits and Compensation

You may benefit from a shorter treatment time and fewer visits with digital and AI-assisted technology;

Your treatment plan will be consistent with conventional orthodontic standards, ensuring treatment efficacy will not be compromised;

The study results may contribute to improving the efficiency and quality of future orthodontic treatment;

Any additional examination costs will be covered by the project; transportation costs for follow-up visits may be reimbursed.

VII. Voluntary Participation and Withdrawal

Participation in this study is voluntary. You may refuse to participate or withdraw at any time. You will not be discriminated against, treated unfairly, or retaliated against for doing so. Your medical care and rights will not be affected.

VIII. Confidentiality of Personal Information

All information collected during the study will be kept confidential and managed by the investigators. Within the limits of the law, research staff, ethics committee members, and relevant authorities may review your records. In any research reports or publications related to this project, your personal identity will not be disclosed.

IX. Contact Information

You may ask any questions about this study at any time. You may contact your physician: Xie Xiaoting, Tel: 13458223809. If you have any complaints about your participation, please contact the Ethics Committee of the Affiliated Hospital of North Sichuan Medical College (Tel: 0817-3371779).

Consent Statement

I have carefully read the above description of this study and have had the opportunity to discuss it with my doctor and ask questions. All my questions have been satisfactorily answered.

I understand that participation is voluntary, and I confirm that I have had sufficient time to consider this decision. I understand that:
(1) I have been informed of the potential risks of participating in this trial and how they will be managed.
(2) I have been informed of alternative treatment options.
(3) I may consult my doctor at any time for more information.
(4) I may withdraw from the study at any time without discrimination or retaliation, and my medical care and rights will not be affected.

I hereby agree to participate in this study and am willing to cooperate with my doctor to complete the research according to the protocol.

Participant signature: _______ Date: _______ Phone: _______
Guardian signature: _______ Date: _______ Phone: _______

I have fully explained to this participant the purpose and procedures of this study, as well as the potential risks and benefits of participation, and have satisfactorily answered all of the participant’s questions.

Investigator signature: _______ Date: _______ Phone: _______

VIII. Research Participants

List of Clinical Trial Investigators

Name	Gender	Title	Education	Specialty	GCP Training	Study Role	Signature
Liu Ying	Female	Professor	PhD	Stomatology	Yes	Principal investigator; overall academic and administrative responsibility; protocol oversight; progress/quality/cost control	
Xie Xiaoting	Male	Lecturer	PhD	Stomatology	Yes	Clinical coordinator; outpatient workflow management; complex case discussions; clinical quality control	
Wu Jianrong	Male	Head Nurse	Master’s	Stomatology	Yes	Responsible for randomization and statistical management; generation of block randomization sequence; review of statistical plan	
Fu Yan	Female	Head Nurse	Master’s	Stomatology	Yes	Responsible for allocation concealment and envelope management; randomization record keeping; monitoring	
Mi Yanxiang	Female	Head Nurse	Master’s	Stomatology	Yes	Responsible for subject recruitment coordination; establishing screening log; initial case reporting	
Liu Si	Female	Head Nurse	Master’s	Stomatology	Yes	Responsible for informed consent processing, file management, ethics submission, and complaint handling	
Li Na	Female	Associate Professor	PhD	Stomatology	Yes	Baseline evaluation and intraoral examination; medical history collection; CRF completion	
Qiong Xian	Female	Lecturer	PhD	Radiology	Yes	Imaging lead; CBCT and 2D imaging collection; quality control and archiving	
Pang Yuhong	Female	Nurse	Bachelor’s	Stomatology	Yes	Digital model scanning process, intraoral STL data management, quality control	

Wu Zengbo	Male	Associate Professor	PhD	Stomatology	Yes	AI-assisted treatment planning, auxiliary measurement and modeling, output of trial versions	
He Huan	Female	Nurse	Bachelor’s	Stomatology	Yes	CAD/CAM and 3D printing lead; retainer design, fabrication and delivery coordination	
Chen Dengen	Male	Associate Professor	Master’s	Stomatology	Yes	Digital–AI group clinical lead; responsible for intervention implementation and follow-up	
Man Yan	Female	Associate Chief Physician	Master’s	Stomatology	Yes	Conventional group clinical lead; responsible for conventional treatment and documentation	
Liu Hua	Female	Lecturer	Master’s	Stomatology	Yes	Occlusal evaluation assessor A; independent occlusal measurement and verification	
Wang Weili	Female	Lecturer	Master’s	Stomatology	Yes	Occlusal evaluation assessor B; occlusal data review and verification	
Guo Yan	Female	Lecturer	Master’s	Stomatology	Yes	Occlusal evaluation assessor C; cross-checking and consistency reporting	
Zhu Wanchun	Male	Associate Professor	PhD	Stomatology	Yes	Data management lead; CRF verification, double entry, data cleaning	
Shi Min	Female	Lecturer	PhD	Stomatology	Yes	Biostatistics and analysis; execution of SAP; regression and multiple corrections	
Huang Dou	Male	Nurse	Bachelor’s	Stomatology	Yes	Adverse event/safety officer; AE/SAE determination and reporting; safety documentation	
Qing Song	Male	Associate Professor	PhD	Stomatology	Yes	Quality control and supervision; SOP compliance audits; CAPA corrective actions	
