# Supplementary material for: Randomized controlled trial comparing AI-assisted digital and conventional orthodontics: Superior PAR reduction and occlusal outcomes
Source: PLoS One. 2026 May 4;21(5):e0347499. doi: 10.1371/journal.pone.0347499 (PMC13138670; doi:10.1371/journal.pone.0347499)
Supplement: S1 Data — Schematic illustration of the five components of the Peer Assessment Rating (PAR) index used to evaluate occlusal outcomes. (A) Upper anterior alignment and (B) lower anterior alignment represent crowding or spacing of the maxillary and mandibular anterior segments, respectively. (C) Overjet reflects the anteroposterior relationship of the anterior teeth. (D) Overbite/open bite depicts the vertical relationship of the anterior teeth. (E) Buccal occlusion illustrates posterior occlusal relationships, incorporating anteroposterior, vertical, and transverse dimensions. S2 Fig. Scatter plots of baseline versus post-treatment PAR scores at T2. Each dot represents an individual participant. Regions indicate categories of change (worse/no difference, improved, greatly improved). Most participants in the Digital and AI group clustered within the “greatly improved” region, whereas those in the Conventional group were more widely distributed across the “partial improvement” and “no improvement” categories. S1 File. Study protocol. S2 File. CONSORT 2010 checklist of information to include when reporting a randomised trial. (ZIP) [file pone.0347499.s001.zip › 04 2 File‎.pdf]

CONSORT 2010 checklist of information to include when reporting a randomised trial*

Section/Topic	Item No	Checklist item	Reported on section No
**Title and abstract**			
	1a	Identification as a randomised trial in the title	Title
	1b	Structured summary of trial design, methods, results, and conclusions (for specific guidance see CONSORT for abstracts)	Abstract
**Introduction**			
Background and objectives	2a	Scientific background and explanation of rationale	Introduction
	2b	Specific objectives or hypotheses	Introduction
**Methods**			
Trial design	3a	Description of trial design (such as parallel, factorial) including allocation ratio	Methods – Study design
	3b	Important changes to methods after trial commencement (such as eligibility criteria), with reasons	N/A
Participants	4a	Eligibility criteria for participants	Methods – Participants
	4b	Settings and locations where the data were collected	Methods – Participants
Interventions	5	The interventions for each group with sufficient details to allow replication, including how and when they were actually administered	Methods – Interventions
Outcomes	6a	Completely defined pre-specified primary and secondary outcome measures, including how and when they were assessed	Methods – Outcome measures
	6b	Any changes to trial outcomes after the trial commenced, with reasons	N/A
Sample size	7a	How sample size was determined	Methods – Sample size estimation
	7b	When applicable, explanation of any interim analyses and stopping guidelines	N/A
Randomisation:			
Sequence generation	8a	Method used to generate the random allocation sequence	Methods – Randomization

	8b	Type of randomisation; details of any restriction (such as blocking and block size)	Methods – Randomization
Allocation concealment mechanism	9	Mechanism used to implement the random allocation sequence (such as sequentially numbered containers), describing any steps taken to conceal the sequence until interventions were assigned	Methods – Randomization
Implementation	10	Who generated the random allocation sequence, who enrolled participants, and who assigned participants to interventions	Methods – Randomization
Blinding	11a	If done, who was blinded after assignment to interventions (for example, participants, care providers, those assessing outcomes) and how	Methods – Randomization
	11b	If relevant, description of the similarity of interventions	N/A
Statistical methods	12a	Statistical methods used to compare groups for primary and secondary outcomes	Methods – Statistical analysis
	12b	Methods for additional analyses, such as subgroup analyses and adjusted analyses	Methods – Statistical analysis
**Results**			
Participant flow (a diagram is strongly recommended)	13a	For each group, the numbers of participants who were randomly assigned, received intended treatment, and were analysed for the primary outcome	Results – 3.1 Participant flow
	13b	For each group, losses and exclusions after randomisation, together with reasons	Results – 3.1 Participant flow
Recruitment	14a	Dates defining the periods of recruitment and follow-up	Methods – Participants; Results – Participant flow
	14b	Why the trial ended or was stopped	N/A
Baseline data	15	A table showing baseline demographic and clinical characteristics for each group	Results – Baseline characteristics
Numbers analysed	16	For each group, number of participants (denominator) included in each analysis and whether the analysis was by original assigned groups	Results – Participant flow
Outcomes and estimation	17a	For each primary and secondary outcome, results for each group, and the estimated effect size and its precision (such as 95% confidence interval)	Results – Primary outcome; Results - Secondary outcomes
	17b	For binary outcomes, presentation of both absolute and relative effect sizes is recommended	Results – Primary outcome

Ancillary analyses	18	Results of any other analyses performed, including subgroup analyses and adjusted analyses, distinguishing pre-specified from exploratory	Results – Subgroup analysis; Results - Multivariable regression
Harms	19	All important harms or unintended effects in each group (for specific guidance see CONSORT for harms)	Results – Adverse events (Section 3.8)
**Discussion**			
Limitations	20	Trial limitations, addressing sources of potential bias, imprecision, and, if relevant, multiplicity of analyses	Discussion – Limitations
Generalisability	21	Generalisability (external validity, applicability) of the trial findings	Discussion – Generalisability
Interpretation	22	Interpretation consistent with results, balancing benefits and harms, and considering other relevant evidence	Discussion – Interpretation
**Other information**			
Registration	23	Registration number and name of trial registry	Methods – Section 2.11 Ethics approval and trial registration
Protocol	24	Where the full trial protocol can be accessed, if available	Supporting Information – Study Protocol (S1 File)
Funding	25	Sources of funding and other support (such as supply of drugs), role of funders	Funding

*We strongly recommend reading this statement in conjunction with the CONSORT 2010 Explanation and Elaboration for important clarifications on all the items. If relevant, we also recommend reading CONSORT extensions for cluster randomised trials, non-inferiority and equivalence trials, non-pharmacological treatments, herbal interventions, and pragmatic trials. Additional extensions are forthcoming: for those and for up to date references relevant to this checklist, see www.consort-statement.org.
